# Dual band Notched 2-port UWB MIMO antenna reconfiguration using lumped capacitors

**DOI:** 10.1038/s41598-026-35976-7

**Published:** 2026-02-05

**Authors:** Wael Ali, Mohamed Abdel Azeem

**Affiliations:** Department of Electronics & Communications Engineering, College of Engineering and Technology, Arab Academy for Science, Technology and Maritime Transport (AASTMT), Alexandria, Egypt

**Keywords:** WLAN, WiMAX, MIMO antenna, Reconfigurable band-notched antenna, Engineering, Physics

## Abstract

In this paper, a dual port reconfigurable band-notched UWB MIMO antenna is presented to reduce the interference with WLAN and WiMAX applications. The suggested MIMO antenna is designed on RO 4350 substrate with partial ground plane for achieving the UWB frequency range with a band-notch at 5.4 GHz to avoid interference with WLAN application. An isolation structure is merged with the partial ground plane to achieve an isolation better than 17 dB between the two elements. Two pairs of lumped capacitors are embedded in the band stop resonators for achieving frequency reconfigurability by shifting the band stop behavior from 5.4 GHz to 3.5 GHz for interference mitigation with WiMAX application. The suggested MIMO antenna is experimentally fabricated and tested to validate the obtained simulated results since a good consistency between both results is achieved not only the impedance characteristics, but also the radiation and diversity outcomes. The new contribution of presented MIMO antenna is evident when it compared with state-of-the-art antennas which confirms the ability of the fabricated model to be utilized for various wireless communication applications in the microwave frequency range.

## Introduction

Microstrip antenna is considered a prominent part in all wireless devices for electromagnetic waves transmission and reception efficiently in terms impedance and radiation characteristics^1^. Microstrip antenna has various advantages when compared to other conventional antennas such as durability, miniaturized size, less complexity, low profile, ease of integration, light weight, and conformal layout on various structures^2^. Several methods are employed to design patch antennas, including: Transmission Line Model which treats the patch as a transmission line shorted at one end, Cavity Model which considers the patch as the top wall of a cavity resonator, and Full-Wave Analysis which employes various numerical methods such as finite element method (FEM) or finite difference time domain (FDTD) to solve Maxwell’s Eq^3^. Due to the on-demand use of high-definition videos and extensive multimedia applications, there are an urgent need greater data rates to fulfill the requirements of these applications. This can be accomplished by the adoption of the simultaneous utilization technique of antenna elements as transmitting and receiving elements which is called multi-input-multi-output (MIMO) technique^4^. The design of MIMO antenna has many challenges especially when the designers are constrained with limited area for the antenna which in turn reduces the spacing between antenna elements.

This reduction in area causes another problem called mutual coupling (electromagnetic interactions between closely separated elements) which degrades the overall performance of the antenna especially impedance matching, diversity and radiation performance^5^. Therefore, an urgent solution for closely separated elements should be introduced for MIMO antenna and this can be achieved by applying various isolation techniques^6^. Various techniques have been reported to increase the isolation between elements by using electromagnetic band gap (EBG)^7^, multimode resonators (MMR)^8^, metamaterial^9^, Neutralization Line (NL)^10,11^ and defected ground structure (DGS)^12^. In^7^, the authors designed a 4-port MIMO antenna to operate at 28 and 38 GHz with EBG structures between radiators for isolation enhancement reaching to values above 25 dB. In order to reduce the mutual coupling between the MIMO elements, MMR structures are utilized in^8^ for orthogonal modes operation (dipole mode and monopole mode), the achieved coupling values are below − 17 dB for 5G MIMO mobile phone antennas. In^9^, a metamaterial array–based 2-port MIMO antenna with dual operating bands 28/38 GHz is introduced. The isolation is enhanced by adding a 3 × 1 metamaterial array between the two radiators by achieving 39/38 dB at 28/38 GHz, respectively. A neutralization line technique can be used to enhance the isolation between the closely separated ports and in^10^ the authors presented a 4-port MIMO antenna for operation in WiFi/WiMAX applications at 2.45/3.5 GHz, respectively. The achieved isolation between ports is better than 25 dB at both bands. Furthermore, the achieved isolation for the case of 2-port 28/38 GHz MIMO antenna in^11^ is above 55 dB between the two ports using NL. The DGS can also be used to improve the isolation between ports as presented in^12^, the MIMO antenna composed of 2 elements and the coupling between ports is better than 40/42 dB at both bands with an achieved gain 6.2/7 dBi, respectively.

Another challenging issue for antenna is to cover the desired frequency band without interfering with the frequency allocated applications such as wireless local area network (WLAN) and worldwide interoperability for microwave access (WiMAX). Consequently, a band-notched antenna needs to be designed in order to mitigate interference with these allocated spectrums.

A notched frequency antenna is a specialized antenna design that intentionally suppresses or attenuates a specific frequency band within its overall operating range. This is achieved by introducing a physical structure, typically a slot or a resonator, into the antenna’s geometry^13^. The underlying physics behind this phenomenon can be explained through the concept of electromagnetic resonance and interference since when the antenna is excited by an electromagnetic wave at its resonant frequency, it absorbs and re-radiates energy efficiently, while when two or more electromagnetic waves interact, they can either reinforce or cancel each other out, depending on their phase relationship^14^.

The mechanism of notch formation is based on the kind of resonator such as slot Resonator where a slot cut into the antenna’s radiating patch which acts as a resonator and at the specific frequency corresponding to the slot’s dimensions, the electromagnetic wave excites the slot, causing it to resonate. The resonant current in the slot interferes destructively with the current on the radiating patch, reducing the antenna’s radiation efficiency at that frequency. Another kind of resonators is the resonant structure such as a stub or a loop that can be added to antenna to absorbs energy at its resonant frequency, reducing the amount of energy available for radiation^15^.

Some antenna designers were interested in designing an UWB antenna with band-notched behaviour and various techniques were utilized to achieve the desired behaviour such as using parasitic elements beside feedline^16^ or beside the partial ground plane^17^. Also, it can be achieved by etching a resonator from the patch^18^ or linking a resonator with the patch^19^ for an improved performance.

A reconfigurable antenna is considered one of the most distinct techniques for efficient spectrum utilization since the user can switch between different frequencies. Moreover, the reconfigurability can extended to radiation and polarization and the user can be capable of switching between even different patterns or polarization modes, respectively^20^. The three different types of reconfigurations can be carried out by using lumped capacitors^16^, using MEMS^21^, using varactor diodes^22,23^, and using PIN diode^24–29^.

A pair of lumped capacitors are used in^16^ in order to switch the notched-band of the UWB antenna from WLAN operated at 5.6 GHz to WiMAX operated at 3.5 GHz. The antenna is designed on FR4 substrate of area 32 × 32 mm^2^ with an average gain of 3 dBi. The authors in^21^ introduced a pattern reconfigurable antenna operated for K-band applications using a pair of RF MEMS switches, the antenna can switch between three modes for three different radiation patterns at 38 GHz for 5G application. In^22^, 4 varactor diodes are embedded on Filtenna to achieve tunable band pass filter (BPF) behaviour (1.75, 2.1, 2.8, and 3 GHz) in the operating band of rectangular microstrip antenna (1.3–3 GHz). Although the reconfigurable antenna achieved the desired performance, but its size isn’t compact enough (80 × 80 mm^2^) to be integrated in hand-held devices.

A dual band 2-port reconfigurable MIMO planar inverted-F antenna (PIFA) is introduced in^23^, 2 pairs of varactor diodes are utilized to reconfigure between two frequency bands (0.9 GHz and 1.7 GHz). The PIFA MIMO achieved a good isolation between the elements better than 20 dB. The authors in^24^ presented a PIN-based folded reconfigurable antenna with multi-sectional configuration to switch between 5 frequency bands (1.45, 1.6, 2, 2.6, and 3.5 GHz) operated in the frequency range (1.5 to 3.5 GHz). The antenna is printed on RO4003 substrate of area 60 × 60 mm^2^ with an improved impedance and radiation characteristics. In^25^, 3 PIN diodes are used to achieved the reconfigurability for the introduced antenna, two PIN diodes for pattern reconfiguration and one for the frequency reconfiguration. The Frequency PIN diode can switch between operation for single band (3.1 GHz) or dual bands (3.1 GHz, 6.8 GHz), whereas the two pattern PIN diodes can switch between 4 different states for the main lobe direction at the two aforementioned frequencies.

In order to achieve the frequency reconfiguration for the suggested MIMO antenna in^26^, a PIN diode is utilized. A decoupling network is employed for achieving band stop behaviour to reduce the mutual coupling between the two elements. The antenna can be switched between two different modes (mode 1 at 4.75 GHz and mode 2 at 1.77 GHz), with isolation of 42.68 dB and 26.52 dB, and gain of 6.63 dBi and 4.41 dBi at both bands, respectively. In^27^, an UWB MIMO is introduced with a reconfigurable band-notched characteristic. The antenna is printed on FR4 substrate of area 50 × 25 mm^2^ for reduced cost purpose with an isolation between ports exceeds 17 dB and an average gain of 3.5 dBi. 4 PIN diodes are utilized to achieve the frequency reconfigurability for the notched frequency band at 4.9–6.3 GHz. In^28^, a reconfigurable MIMO antenna is presented to switch between 4G/LTE and 5G/Sub-6 GHz using 4 PIN diodes. The 2 ports are placed diagonally to each other on 120 × 60 mm^2^ substrate for mutual coupling reduction (≤ 12 dB). The antenna can be reconfigured between 2.4 GHz (2.2 GHz to 2.7) and 3.5 GHz (3.3 GHz to 4.02 GHz) with realized gain 3.7 dBi and 4.2 dBi, respectively. The authors in^29^ suggested a reconfigurable band-notched MIMO antenna for UWB applications. The PIN diodes are used to switch ON/OFF the notched band centered at 5.5 GHz (5.15–5.82 GHz). The isolation between ports is improved due to the utilized decoupling structure which provide an isolation greater than 20 dB between the two ports with realized gain of 1–2.5 dBi. In^30^, 4-port MIMO antenna is introduced with 4-pairs of PIN diodes to achieve three modes of operation inside UWB frequency range. The antenna succeeded to achieve the desired performance such as isolation between ports greater than 18.6 dB and an acceptable average gain of 5.5 dBi over the achieved bands for various states. 3-pairs of PIN diodes are utilized and embedded in 2-port MIMO antenna to achieve 6-modes operated at three notched frequencies (3.5,4.5, 7.5 GHz) in^31^. The gain of MIMO antenna is ranging from 1.15 − 5.23 dBi with an isolation between ports larger than 32 dB.

In this paper, a band-notched 2-port MIMO antenna is designed to operate in the UWB frequency except for WLAN at 5.4 GHz. An isolation structure is implemented on the back side of the antenna and merged with the partial ground planes for mutual coupling reduction between elements. Two pairs of lumped capacitors are utilized to shift the band stop behaviour from 5.4 GHz to 3.5 GHz. The model is experimentally fabricated to validate the obtained results using high frequency structure simulator (HFSS) software and good agreement is evident between simulated and measured outcomes affirming the importance of the suggested antenna for various wireless applications.

The major highlights of the suggested MIMO antenna can be outlined as follows:

- Superior diversity performance: Achieved an exceptionally low envelope correlation coefficient (ECC) less than 0.009. This indicates excellent port isolation and minimal signal correlation, crucial for high-performance MIMO systems.
- High isolation: Demonstrated good isolation with value greater than 17 dB between the two ports.
- Compact and thin design: Achieved a compact size of 40 × 26 mm^2^ with a very thin substrate thickness of 0.8 mm for miniaturization purpose.
- Effective Reconfigurability: Employed 4 lumped capacitors for frequency reconfiguration between WLAN and WiMAX applications, representing a distinct approach for biasing and integration when compared to other complex approaches.
- Acceptable Realized Gain: Achieved realized gain ranging from 1.2 to 4.7 dBi over the achieved frequency band.

## Single antenna layout

**Fig. 1:**
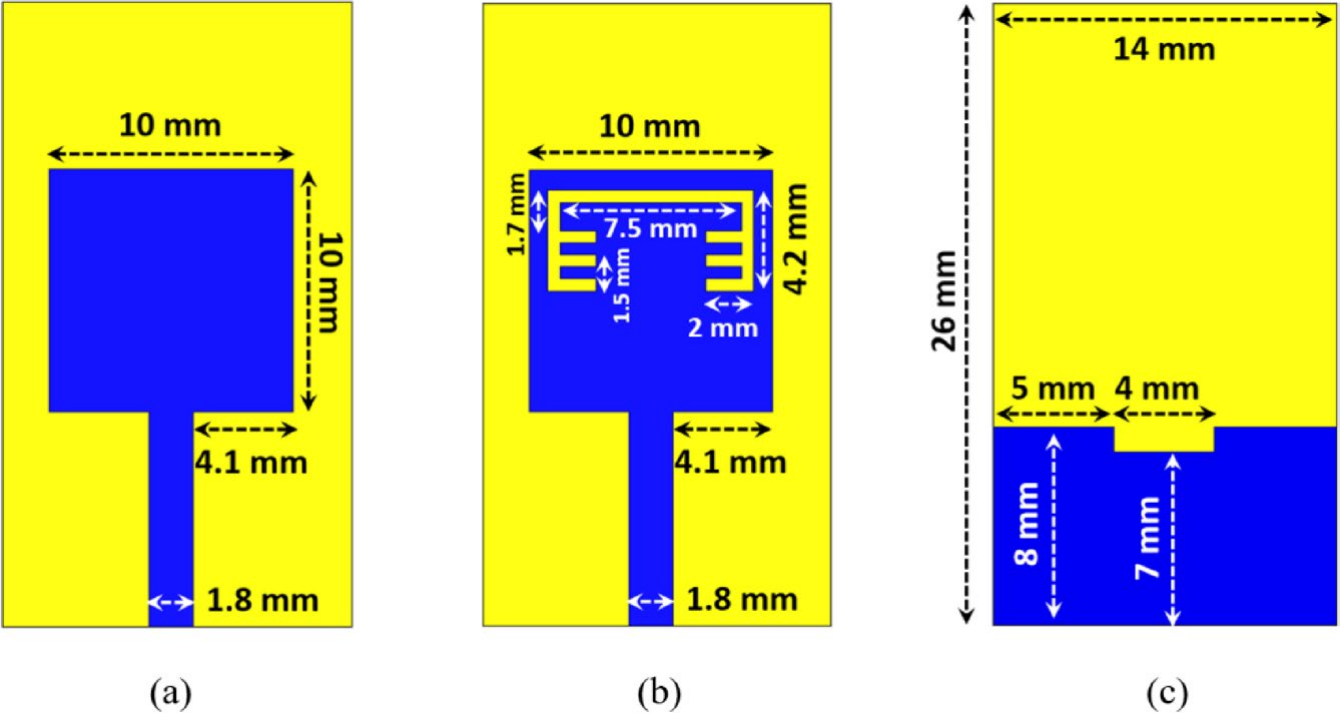
Band-notched UWB microstrip antenna (**a**) Front-view (without notch) (**b**) Front-view (with notch) (**c**) Back-view.

Figure. 1 (a) illustrates the top-view configuration of the reference single element antenna. It is consisted of a 50-ohm microstrip line feed (1.8 mm width and 9 mm length), connected to rectangular patch antenna. The antenna is designed on RO4350 (26 × 14 mm^2^) substrate with dielectric constant of 3.66, loss tangent of 0.004 and height of 0.8 mm. A pair of comb-shaped structure are etched from the antenna structure to achieve the band-notched behaviour as depicted in Figure. 1 (b). A partial ground plane is utilized as shown in Figure. 1 (c) to achieve an UWB behaviour instead of the conventional narrow behaviour of full ground plane for both antennas with/without band-notched structures. The length of etched slot from patch is calculated as /2 where is the guided wavelength at the notched frequency, *f*_*notch*_ is the notched frequency, *ε*_*eff*_ is the effective dielectric constant, and *ε*_*r*_ is the relative dielectric constant^31,32^.

1 $$\:{\lambda\:}_{g}=\frac{c}{{f}_{notch}\sqrt{{\epsilon\:}_{eff}}}$$

2 $$\:{\epsilon\:}_{eff}=\frac{{\epsilon\:}_{r}+1}{2}$$

**Fig. 2:**
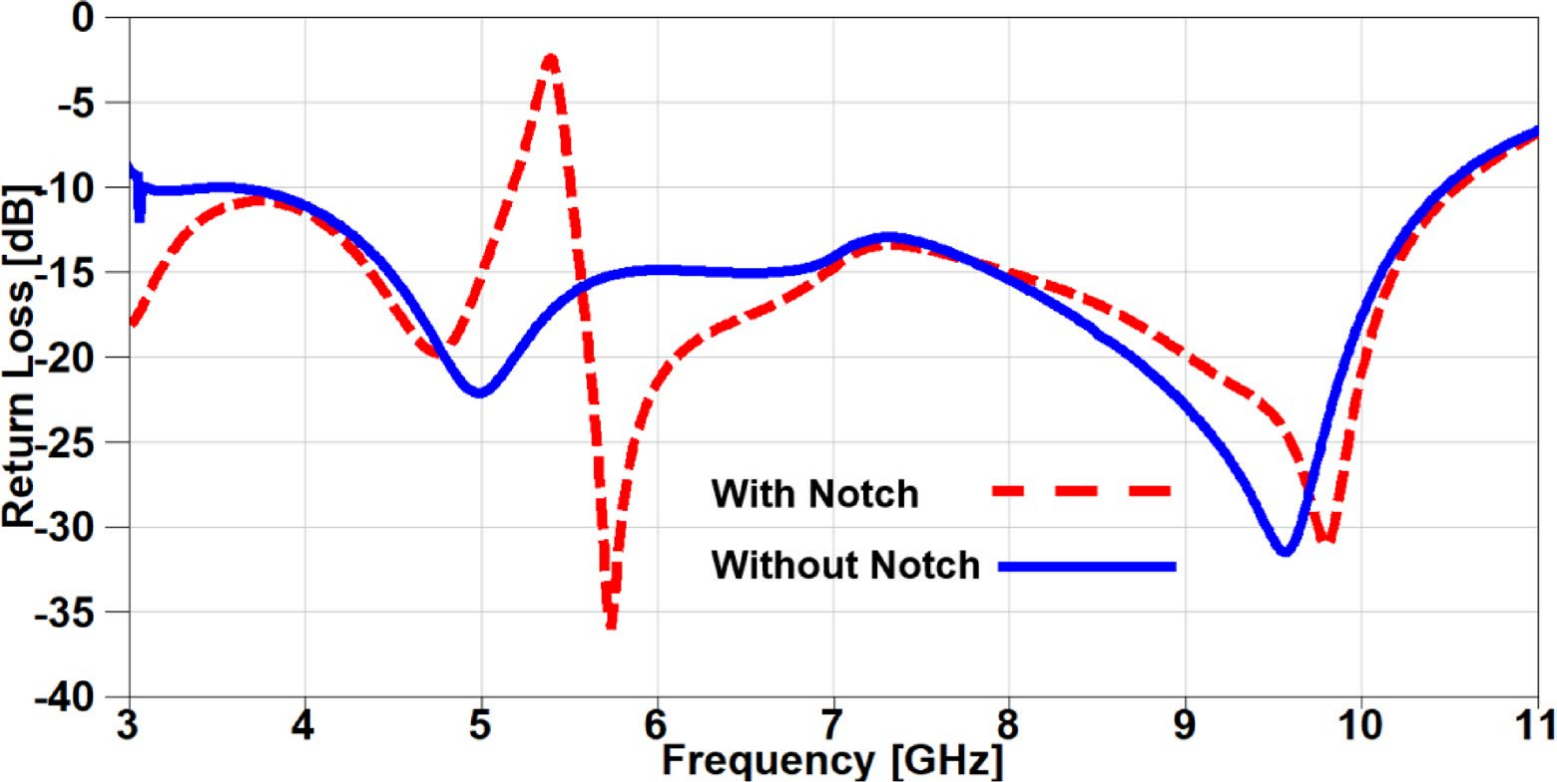
The S_11_ outcomes of UWB antenna with/without band-notched structure.

**Fig. 3:**
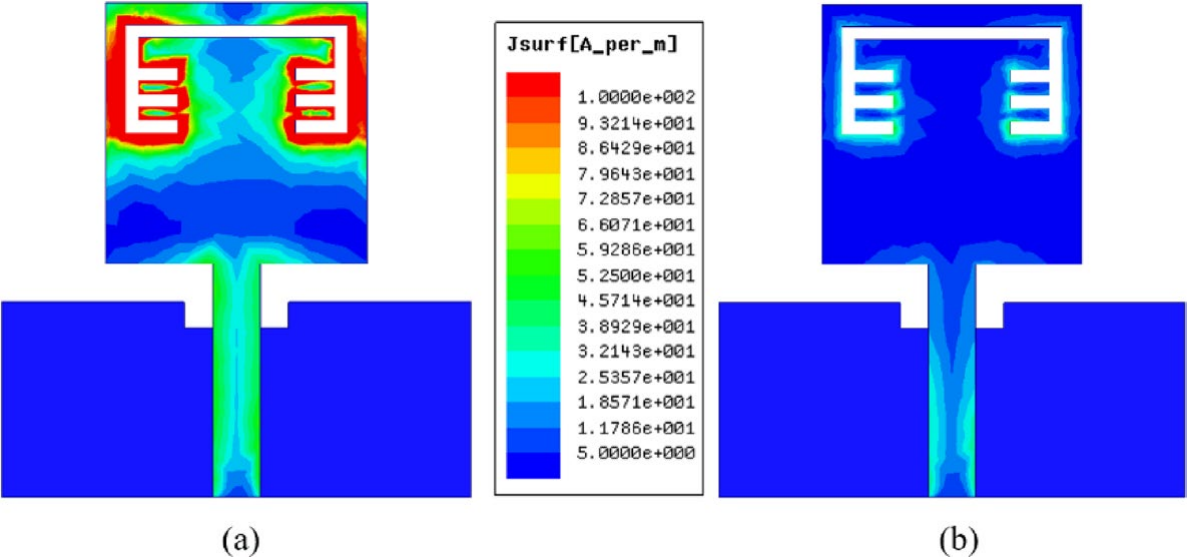
The current distribution of band-notched UWB antenna (**a**) at 5.4 GHz (**b**) at 6 GHz.

The S_11_ outcomes of the antenna are depicted in Figure. 2 for the antenna structure with/without notch and it can be noticed that the behaviour of the antenna is below − 10 dB from 3 GHz to 10.6 GHz for without notch curve, whereas the antenna achieved the frequency band (3–10 GHz) except a band-stop behaviour at 5.4 GHz for the other curve and this is due to the etched structure from the antenna which can be used for interference mitigation with WLAN. In order to investigate the effectiveness of the band-notched structure, a current distribution analysis is carried out on the introduced antenna at two different frequencies inside the achieved frequency band. It can be noticed from Figure. 3 (a) that the current is gathered at the band-stop structure when the applied signal operated at 5.4 GHz (notched-frequency), while the current is normally distributed when it is operated at 6 GHz (outside notched-band) as shown in Figure.3 (b).

## Parametric study on single antenna layout

**Fig. 4:**
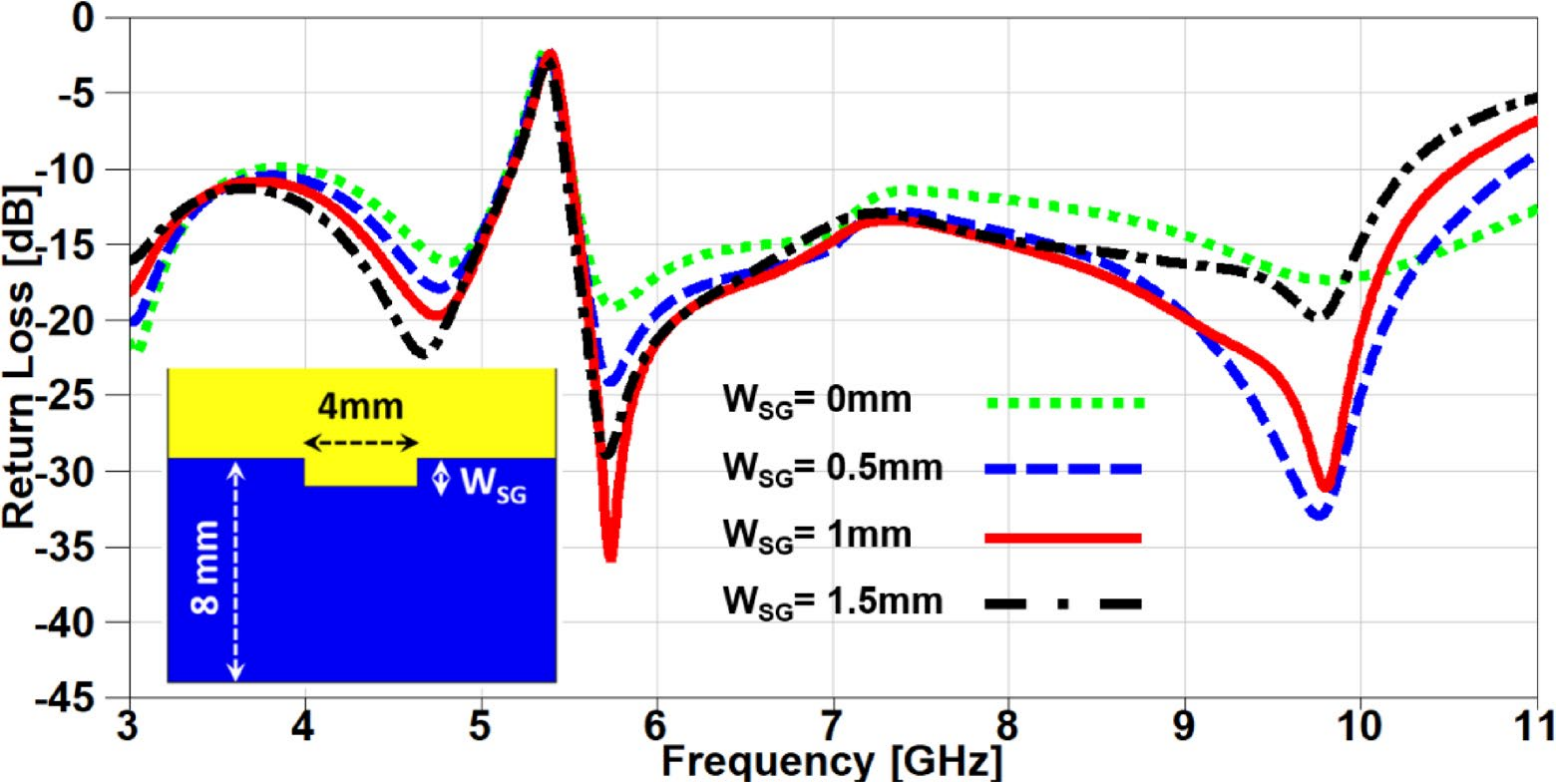
The variation of partial ground slot width on antenna performance.

**Fig. 5:**
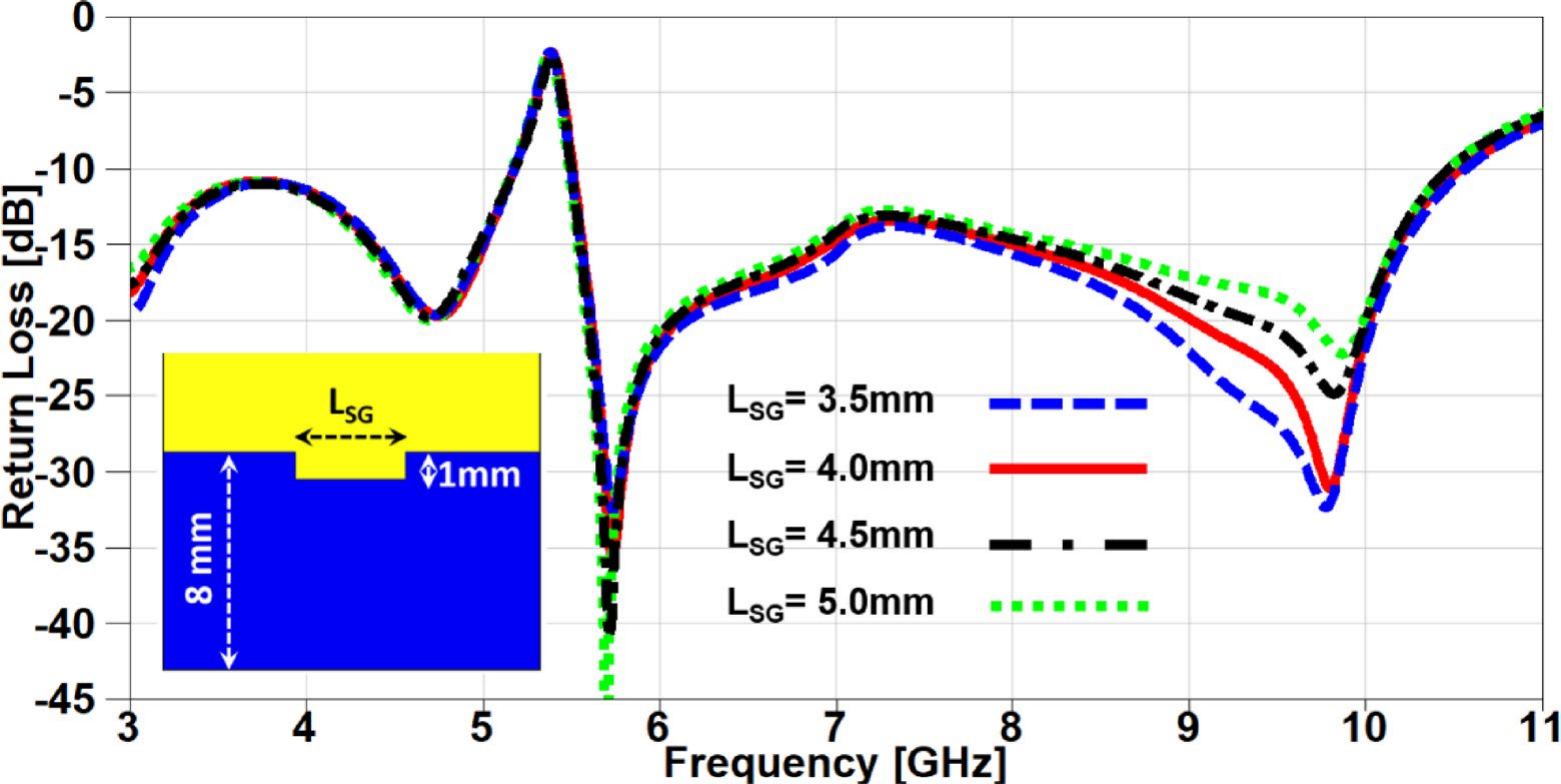
The variation of partial ground slot length on antenna performance.

**Fig. 6:**
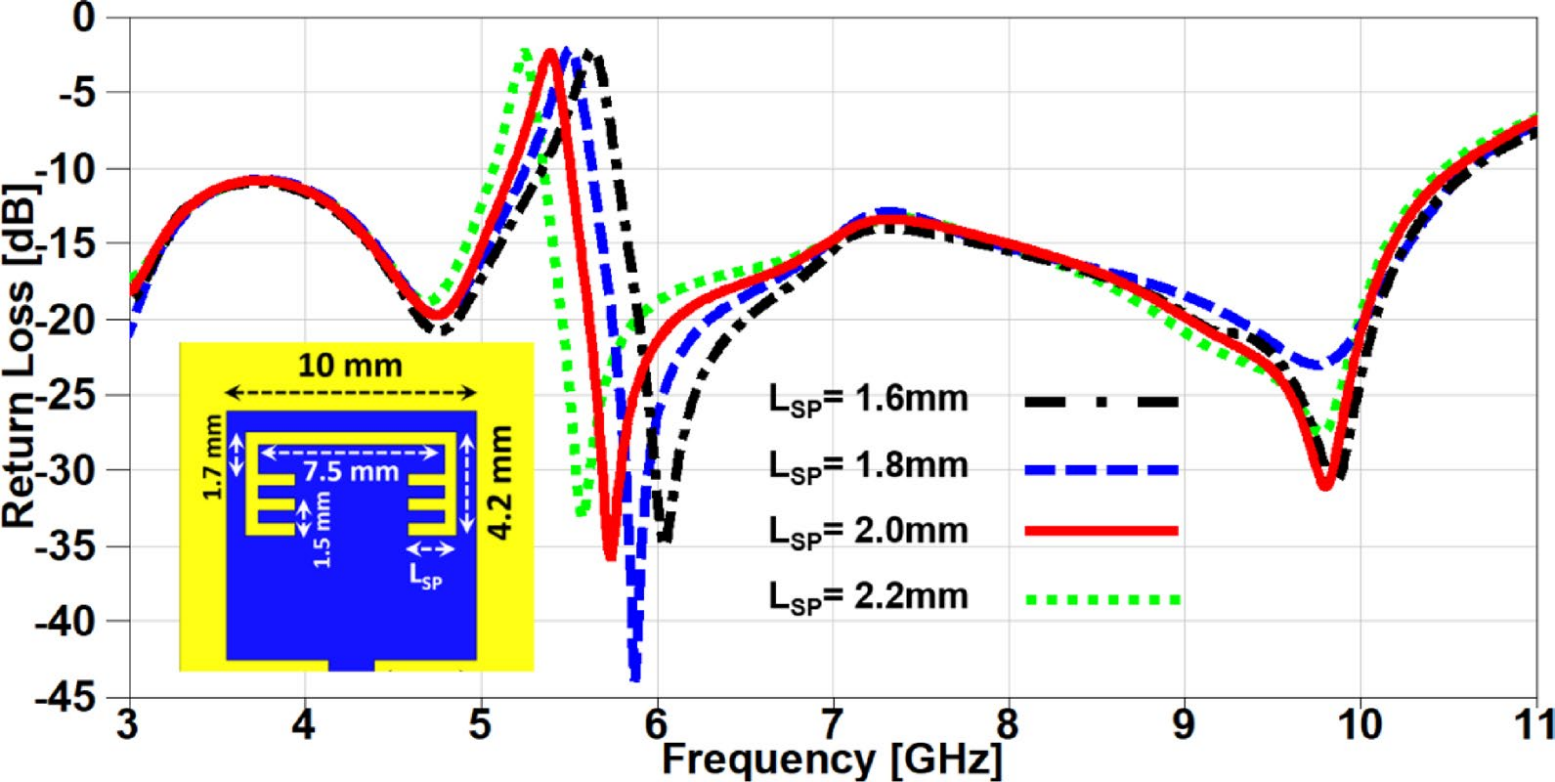
The variation of the patch smaller slots lengths on antenna performance.

**Fig. 7:**
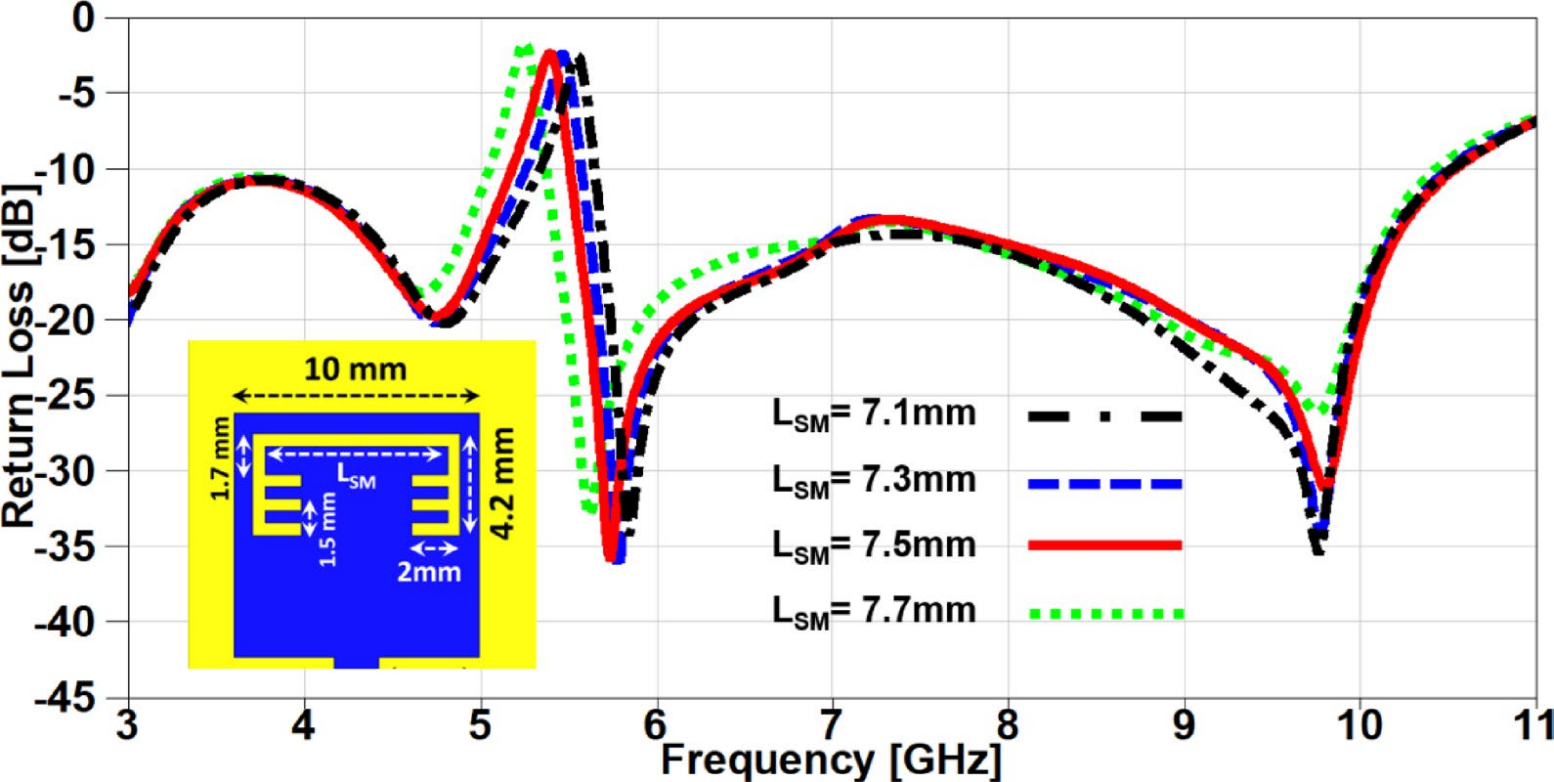
The variation of the patch larger slot length on antenna performance.

In this section, a parametric study on the essential design parameters is carried out. Figure. 4 illustrates the effect of changing the width of partial ground slot (W_SG_) and it can be noticed that increasing the slot width can constructively effect on the UWB behaviour and after 1 mm the performance is degraded especially in the higher frequency range (7–11 GHz). The effect of changing the slot length (L_SG_) is investigated in Figure. 5, there is no significant effect in the lower UWB range (3–7 GHz). on the contrary, the performance is improved in the higher frequency range (7–11 GHz) when the slot length is decreased. In order to study the behaviour of the band-notched structure, the key elements that have significant effects on the notched resonance frequency are investigated as presented in Figs. 6 and 7. The effect of changing smaller slots lengths (L_SP_) is introduced in Figure. 6 and it can be observed that the resonance frequency is increased by decreasing the slot length and this study is carried out to reach the desired notched frequency at 5.4 GHz. Furthermore, the variation of larger slot length (L_SM_) is demonstrated in Figure. 7 and it is clearly noticed that the notched resonance frequency is shifting slightly when the length is increased from 7.1 to 7.5 mm and then it is shifted significantly when it becomes 7.7 mm.

**Fig. 8:**
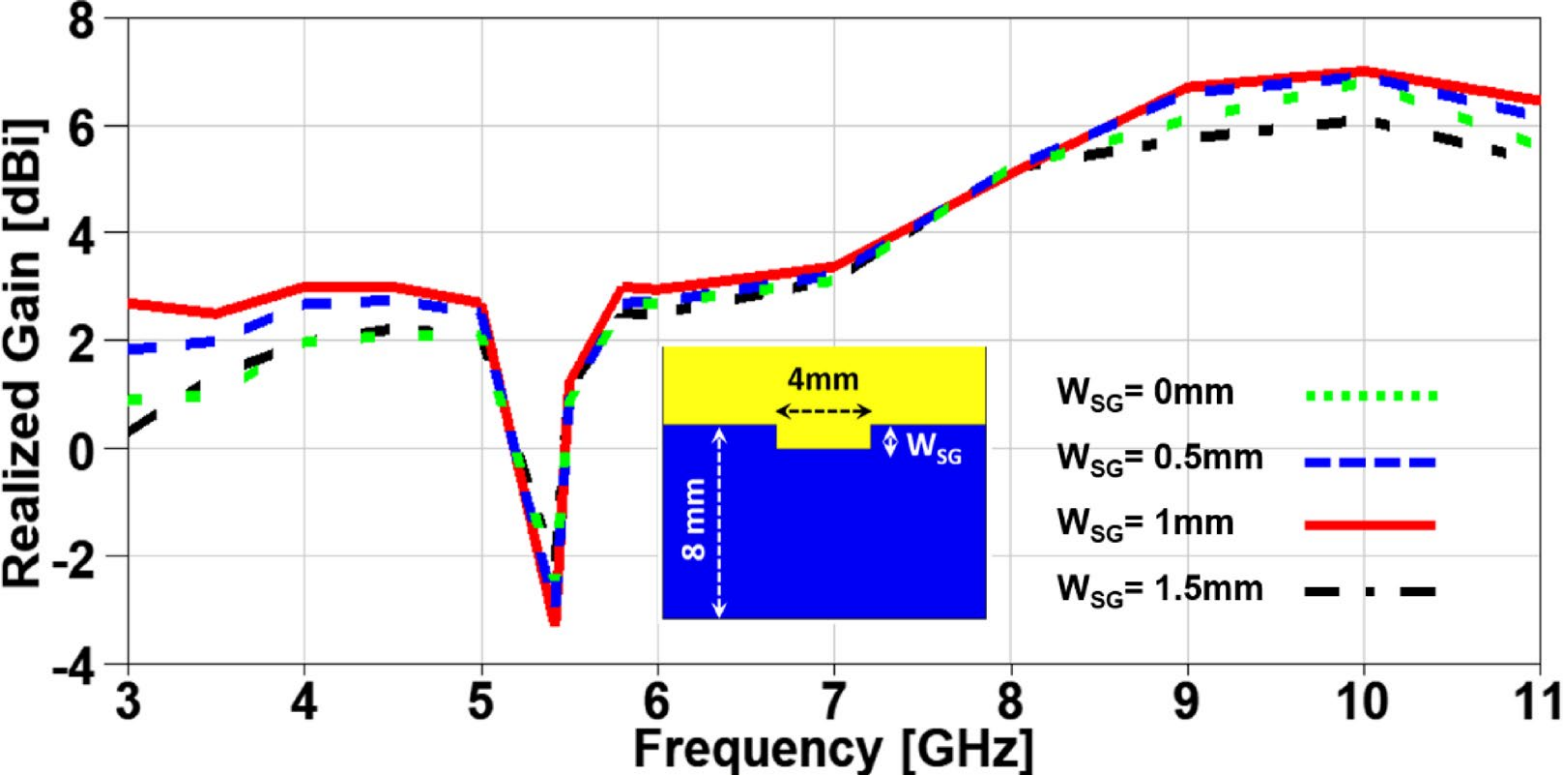
The variation of partial ground slot width on antenna performance.

**Fig. 9:**
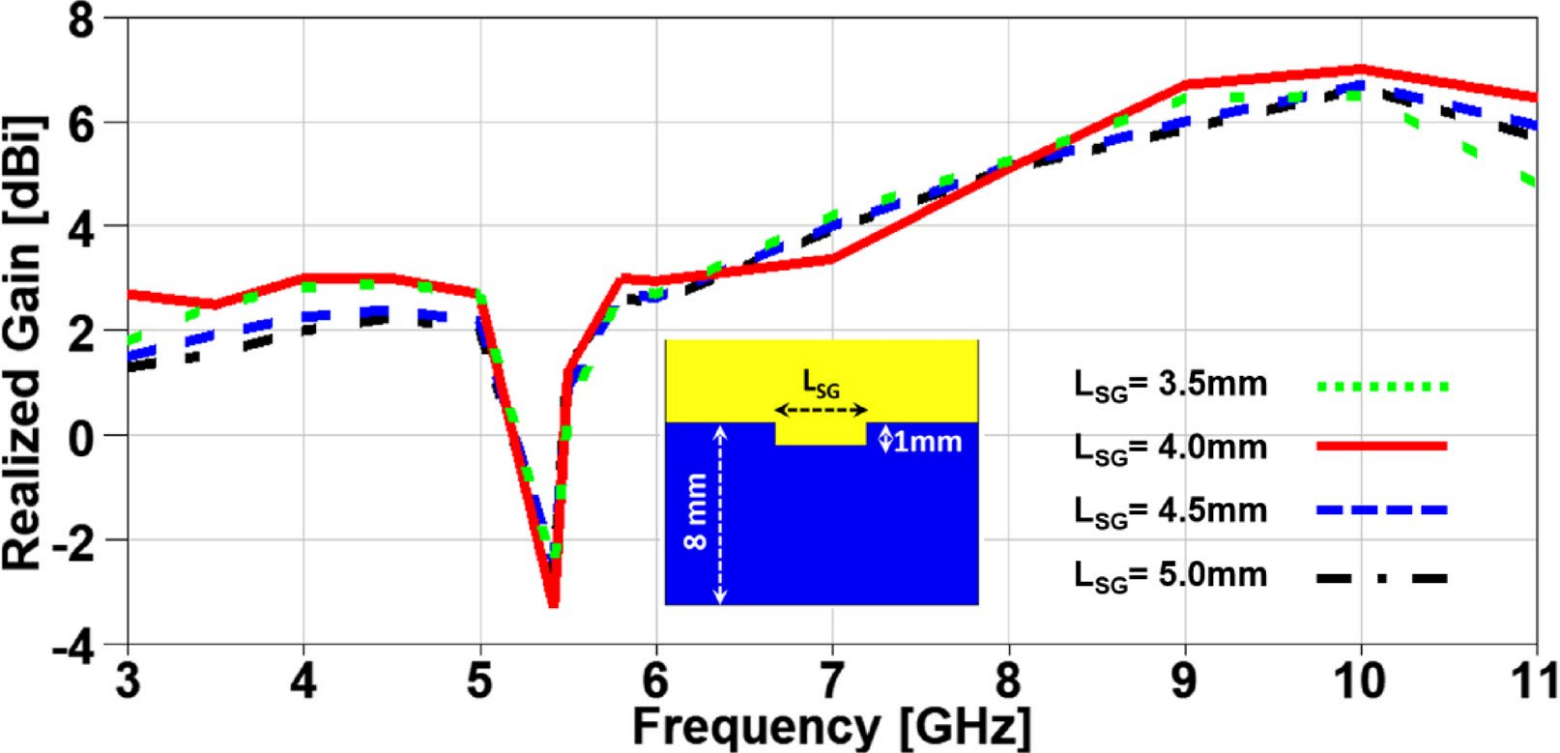
The variation of partial ground slot length on antenna performance.

**Fig. 10:**
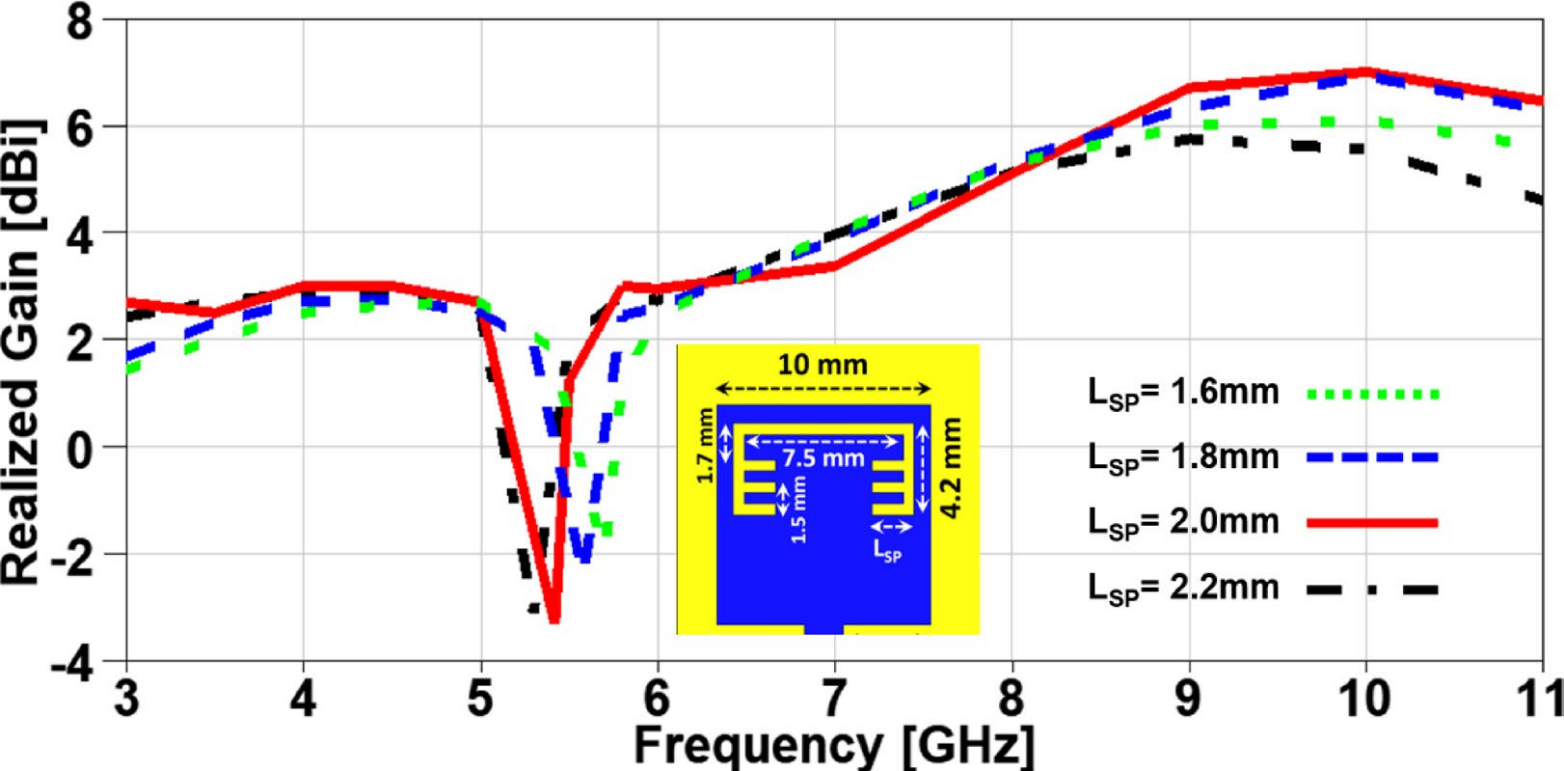
The variation of the patch smaller slots lengths on antenna performance.

**Fig. 11:**
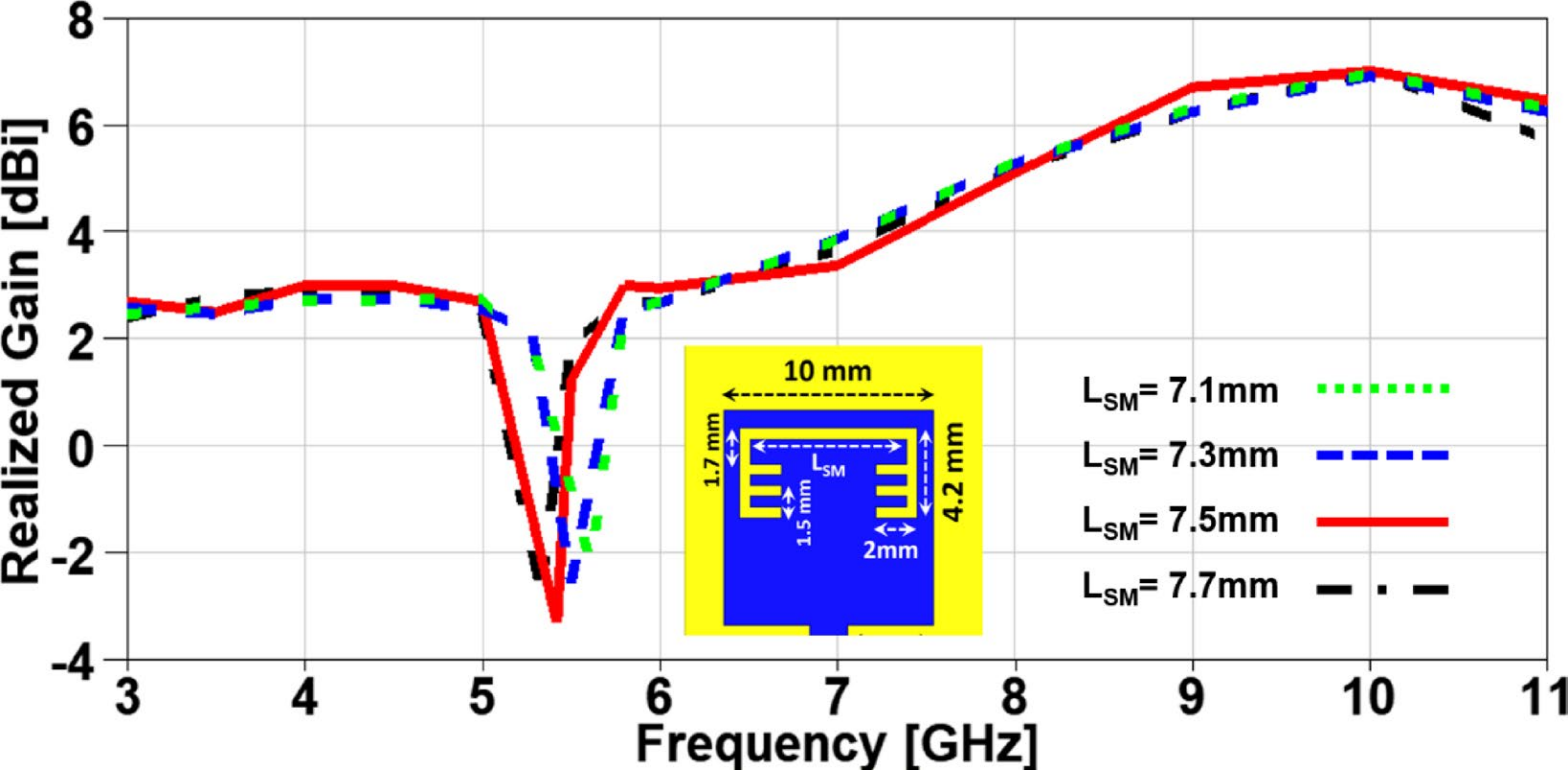
The variation of the patch larger slot length on antenna performance.

In order to investigate the impact of variating the design parameters not only on the impedance characteristics of the suggested MIMO antenna, but also on its radiation characteristics, a parametric study on the aforementioned parameters is carried out with its impact on the realized gain is presented in Figs. 8, 9, 10 and 11. It is clearly noticed from Figure. 8 that the notched band resonance is fixed when is W_SG_ changed, but the best realized gain values are achieved when W_SG_ = 1 mm. Figure. 9 introduces the impact of changing L_SG_ on the realized gain and it can be observed a deeper band notched behavior at 5.4 GHz for the case of L_SG_ = 4 mm with an improved performance over the achieved band except a minor degradation in the range from 6.5 to 8 GHz. The effect of changing L_SP_ can be demonstrated from Figure. 10 where the notched band resonance is significantly shifted from 5.7 GHz to 5.3 GHz when L_SP_ is increased, and the case of L_SP_ showed an improved performance when compared to other cases, except the range from 6.3 to 8.2 GHz. Moreover, the variation of L_SM_ and its impact on the realized gain of the suggested MIMO antenna is depicted in Figure. 11 and it can be seen that the behaviors are approximately the same in the achieved frequency band, but the notch resonance is shifted from 5.61 GHz to 5.33 GHz when L_SM_ is increased. All the previous parameters are investigated to clearly identify the best values design parameters that can produce the desired performance in terms of achieving the UWB frequency range with a band-notched behaviour at 5.4 GHz and higher realized gain over the achieved frequency band for an improved impedance and radiation characteristics, respectively.

### Port MIMO antenna layout

**Fig. 12:**
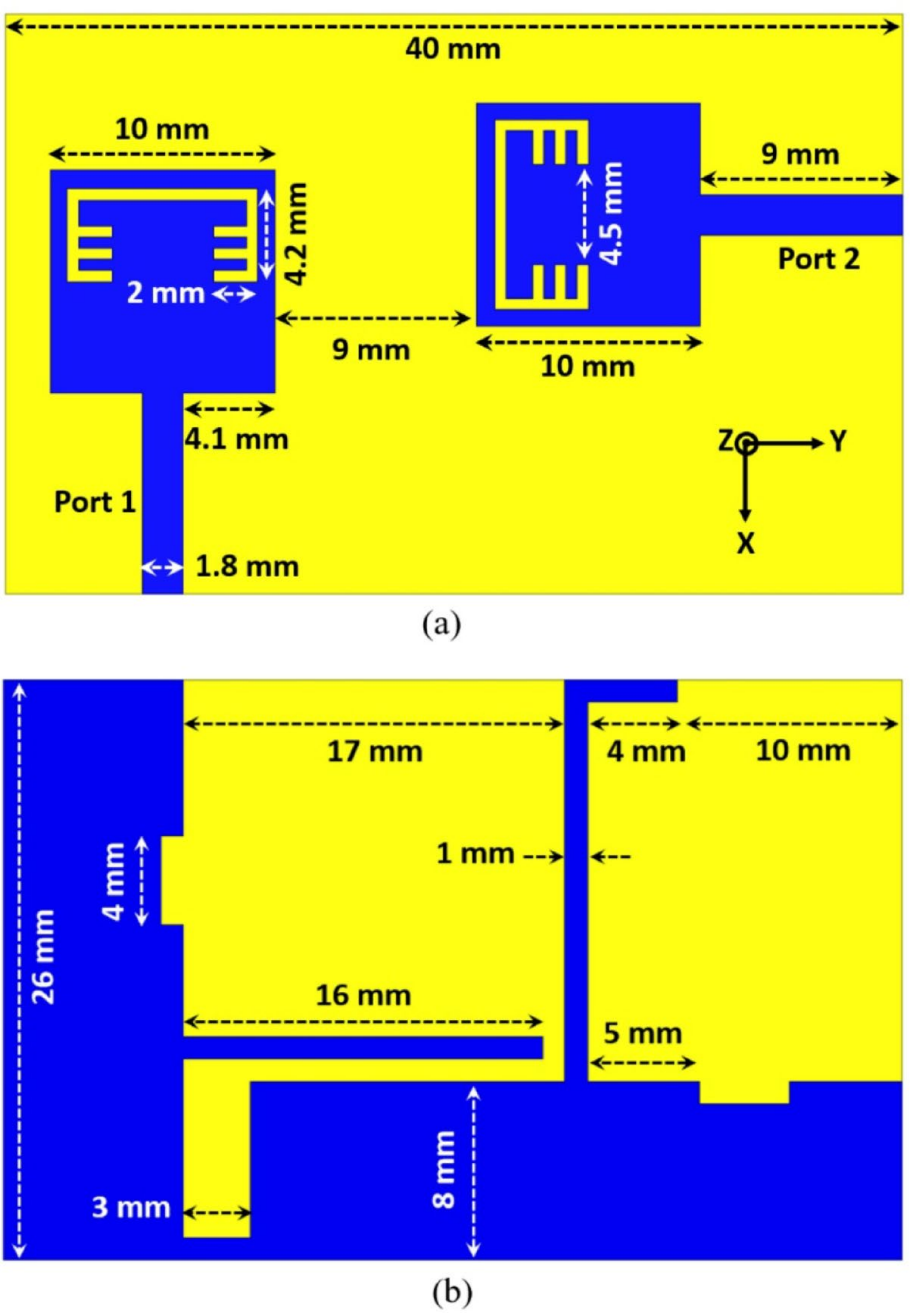
Band-notched 2-port MIMO UWB antenna (**a**) Front view (**b**) Back view.

**Fig. 13:**
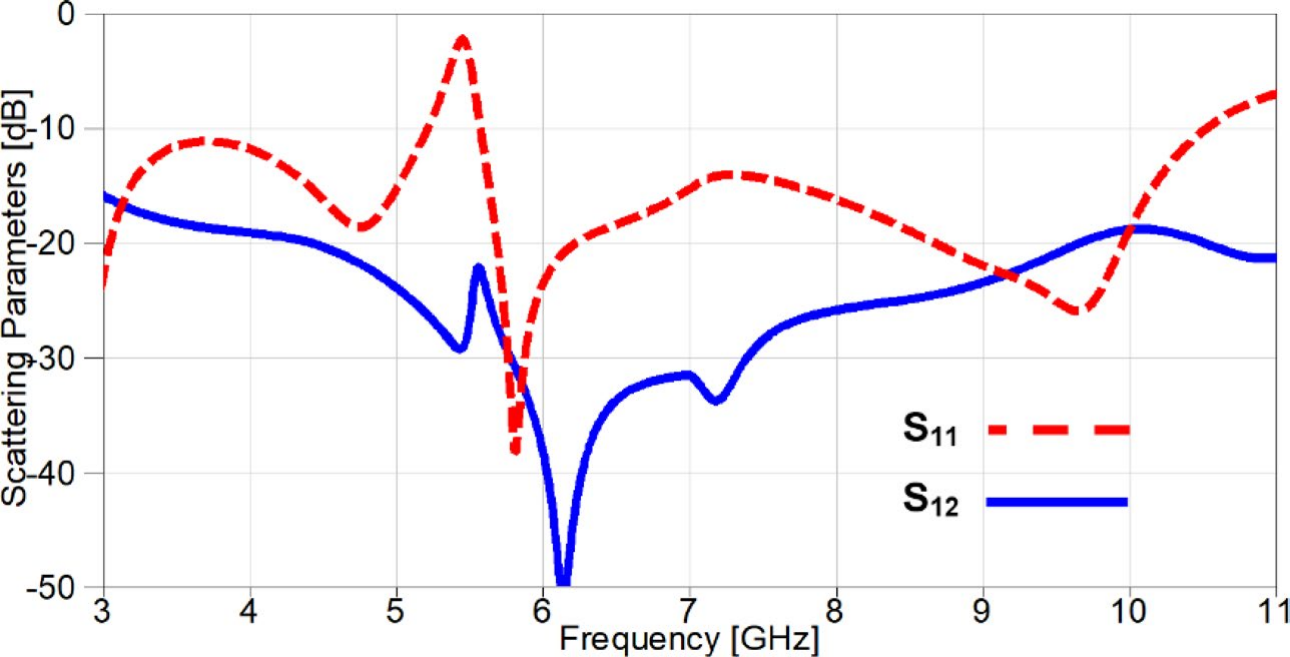
The simulated S_11_ with/without stubs.

In order to construct a MIMO antenna, an additional element of the antenna shown in Figure.1 (b) is added on the same substrate after extending its area to be 40 × 26 mm^2^. The orthogonal arrangement with a spacing of 9 mm between elements is utilized as depicted in Figure. 12 (a) in order to reduce the mutual coupling. Furthermore, the partial grounds in the back side are connected together with two additional rectangular stubs for achieving a good isolation between elements which in turn will affect directly on the diversity behaviour of the MIMO antenna. The obtained results from the MIMO antenna shown in Figure. 12 are simulated using HFSS and it can be observed from Figure. 13 that the antenna covered an UWB frequency range from 3 GHz till 10.6 GHz (below − 10 dB) with a band-notched response at 5.4 GHz (above − 10 dB) confirming the availability of the presented MIMO antenna to reduce interference with frequency allocated application at 5.4 GHz (WLAN). Also, another parameter that can decide the efficacy of the MIMO antenna is the isolation, and it is evident from Figure. 13 that the isolation between elements is greater than 20 dB over the achieved band, which will positively impact on the diversity parameters of the MIMO antenna.

**Fig. 14:**
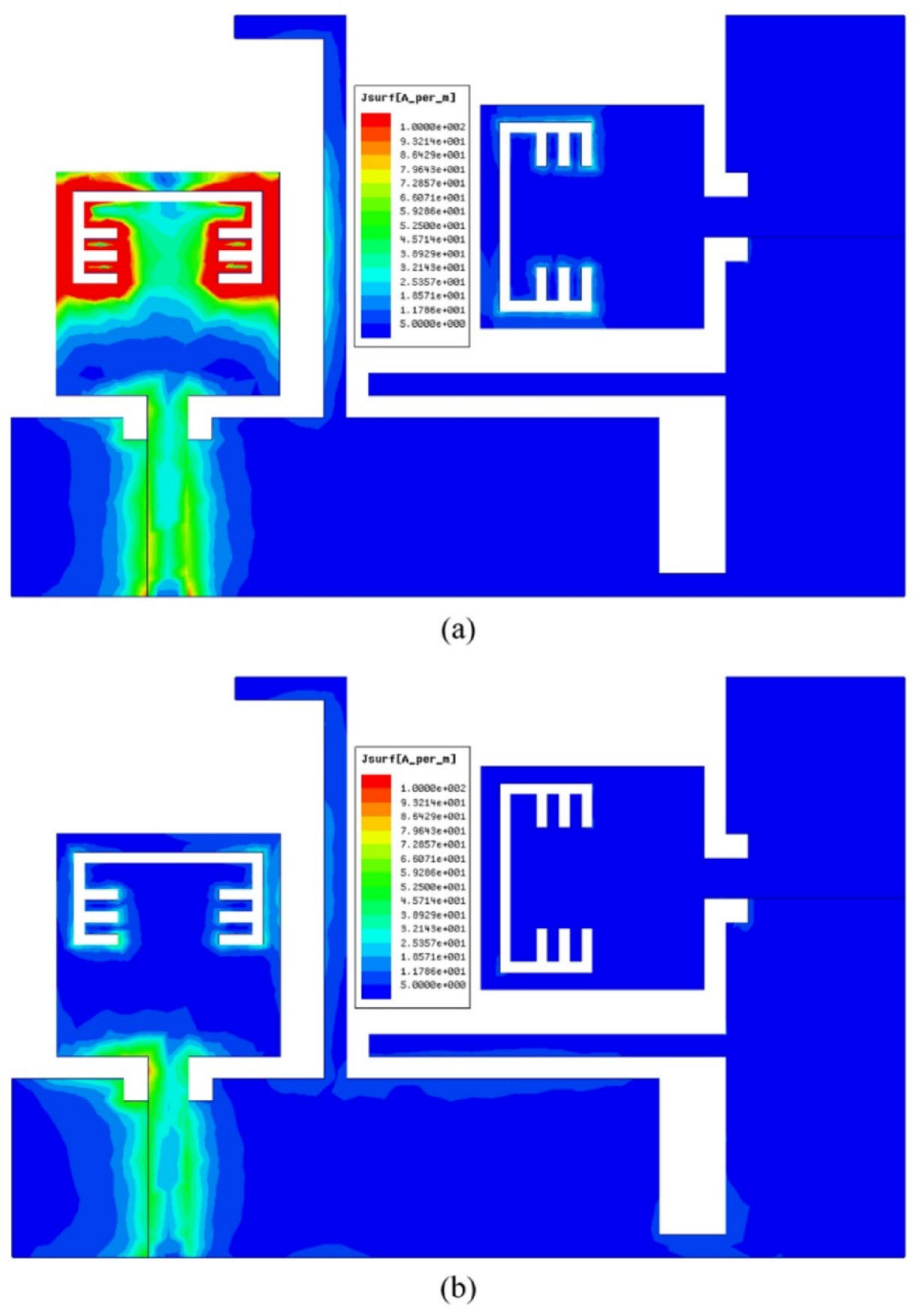
The current distribution of the suggested band-notched MIMO at two different frequencies (**a**) at 5.45 GHz (**b**) at 5.8 GHz.

The current distribution of the suggested MIMO antenna is shown in Figure. 14 for two different frequencies (5.45 GHz inside the notch and 5.8 GHz outside the notch) and it can be observed that the antenna can block the radiation when its port is excited with RF signal of frequency 5.45 GHz since the current is distributed over the band-stop structure as depicted in Figure. 14 (a), also a very small amount of current is transferred to the other port which confirms the low mutual coupling between the two ports and this is due to the integrated stubs with the partial ground planes.

**Fig. 15:**
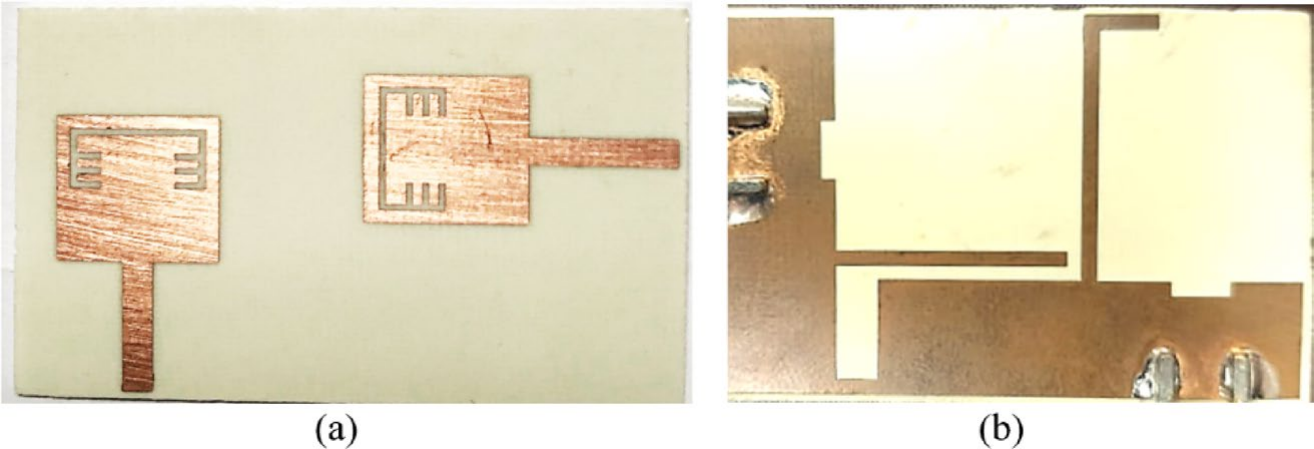
The fabricated photograph of the 2-port MIMO antenna (**a**) Front view (**b**) Back view.

**Fig. 16:**
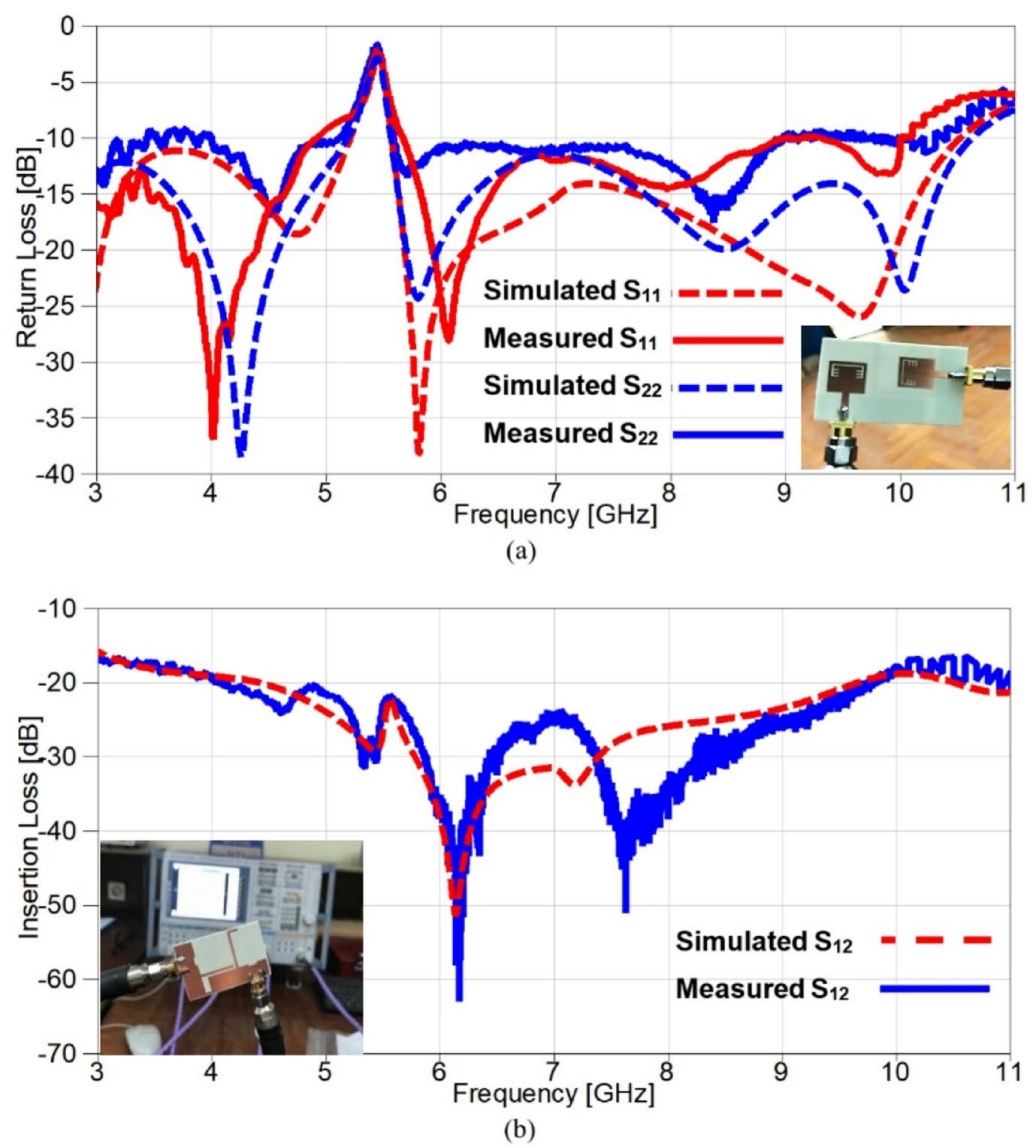
The scattering parameters of the suggested band-notched 2-port MIMO antenna (**a**) Return loss (**b**) Insertion loss.

To validate the obtained simulated outcomes of the suggested UWB band-notched MIMO antenna, the MIMO model is fabricated as shown in Figure. 15 which presents the top and bottom views of the experimentally fabricated model and this model is measured using Rohde & Schwarz ZVA67 vector network analyzer (VNA) with an operating frequency ranging from 10 MHz to 67 GHz. Figure. 16 illustrates the comparison between simulated and measured outcomes for the two ports and it is clear that the measured S_11_ and S_22_ outcomes mimic the simulated ones where both have a band-stop behaviour at 5.4 GHz, also the obtained return loss is below − 10 dB from 3 GHz to 10 GHz for measured results whereas it covers approximately the same band (3–10.6 GHz) for the simulated S_11_ and S_22_ as shown in Figure. 16 (a). Another important factor is also validated to investigate the mutual coupling level between elements and Figure. 16 (b) introduces a comparison between insertion loss results of the proposed antenna. It can be observed that the exemplary agreement between both S_21_ results confirming the eligibility of the band-notched MIMO antenna for modern wireless technologies without interfering applications with allocated spectrum.

**Fig. 17:**
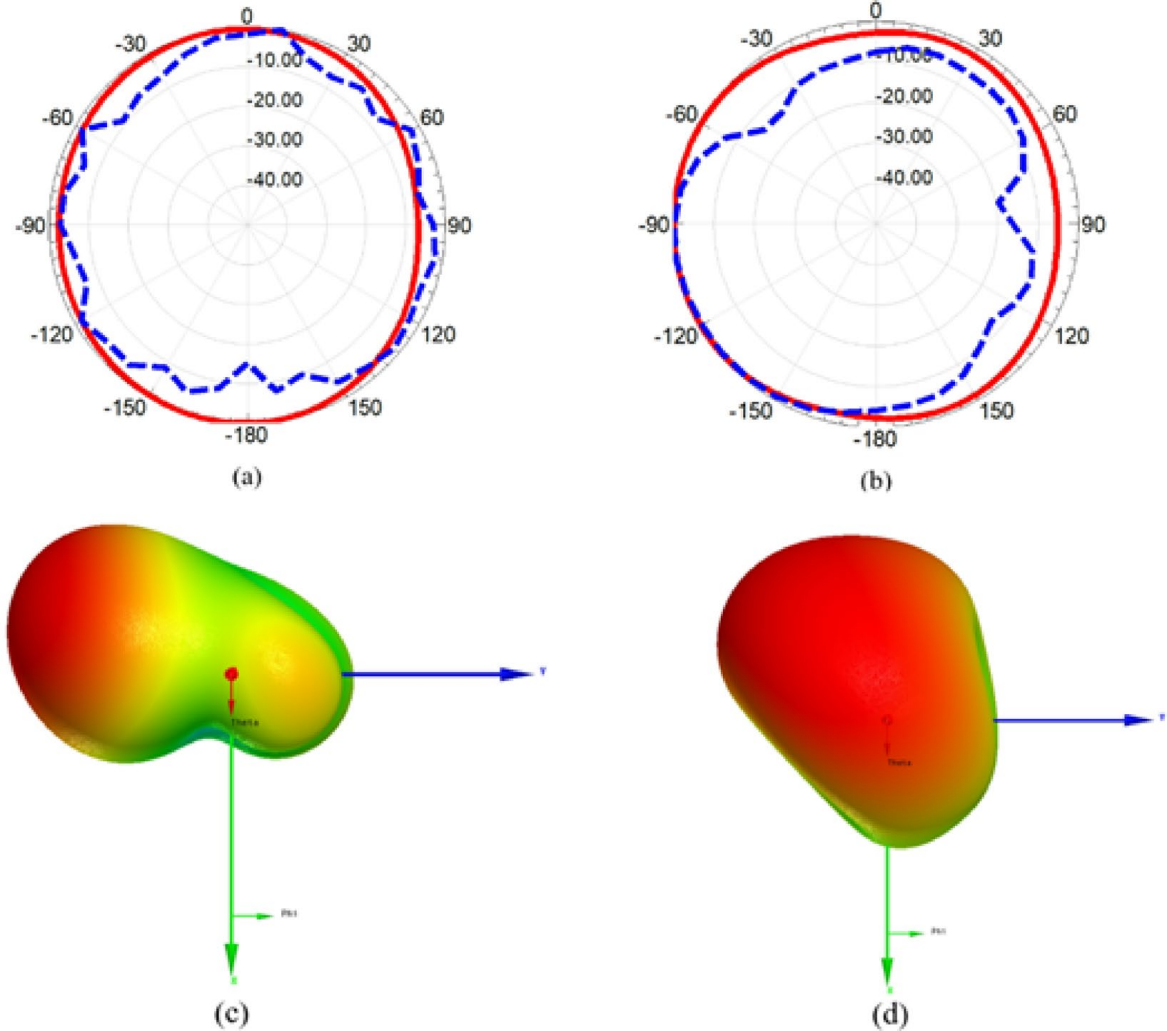
The 2-D simulated (solid) and measured (dashed) radiation patterns, and 3-D radiation pattern of the reconfigurable MIMO antenna at 5.8 GHz (**a**) at x-z plane (**b**) at y-z plane. (**c**) 3-D pattern for port 1 at x-y plane (**d**) 3-D pattern for port 2 at x-y plane.

One of the important parameters needs to be investigated is the radiation pattern which is carried out in anechoic chamber. A reference horn antenna worked at frequency band from 0.7 GHz up to 20 GHz with standard gain values, and is linked to microwave source which is used as transmitting antenna while the suggested MIMO is mounted on the receiving side. The two antennas have a line of sight with 150 cm separation between them and the reflected wave is absorbed by the chamber absorbers. The suggested antenna is rotated to allow real-time radiation pattern measurements. Figure. 17 demonstrates the radiation patterns in both planes (E and H planes) and it is evident from figure that the radiation patterns of the MIMO antenna are nearly omnidirectional in both planes and there is a significant consistency between simulated and measured outcomes. Moreover, the obtained patterns are the most favorable patterns for modern wireless devices. Furthermore, the 3-D radiation patterns in x-y plane for each excited port are shown in the Figure. 17 (c) and (d) and it is evident that the suggested MIMO antenna provides a pattern diversity with an approximate orthogonal beam directions due to the orthogonal arrangement of the two elements confirming the low-level of cross polarization as well as the independency of each element for radiating/receiving EM waves.

**Fig. 18:**
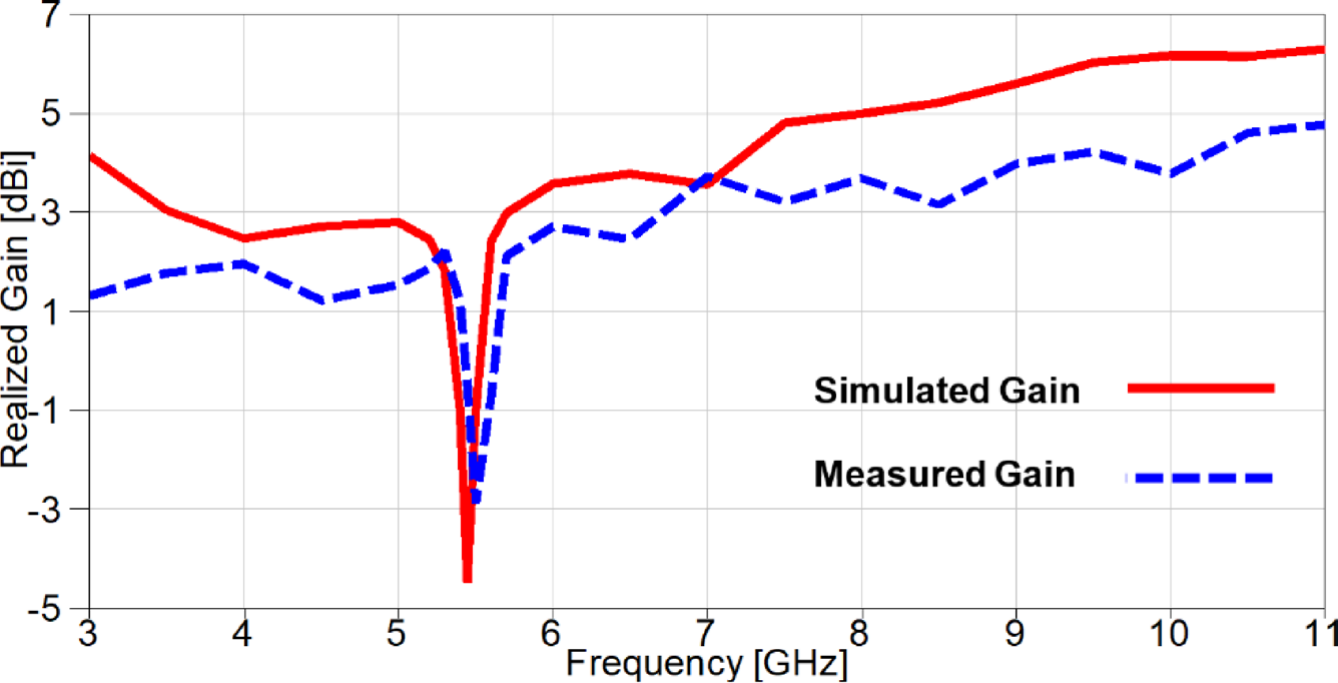
The simulated and measured realized gain of the suggested band-notched MIMO.

**Fig. 19:**
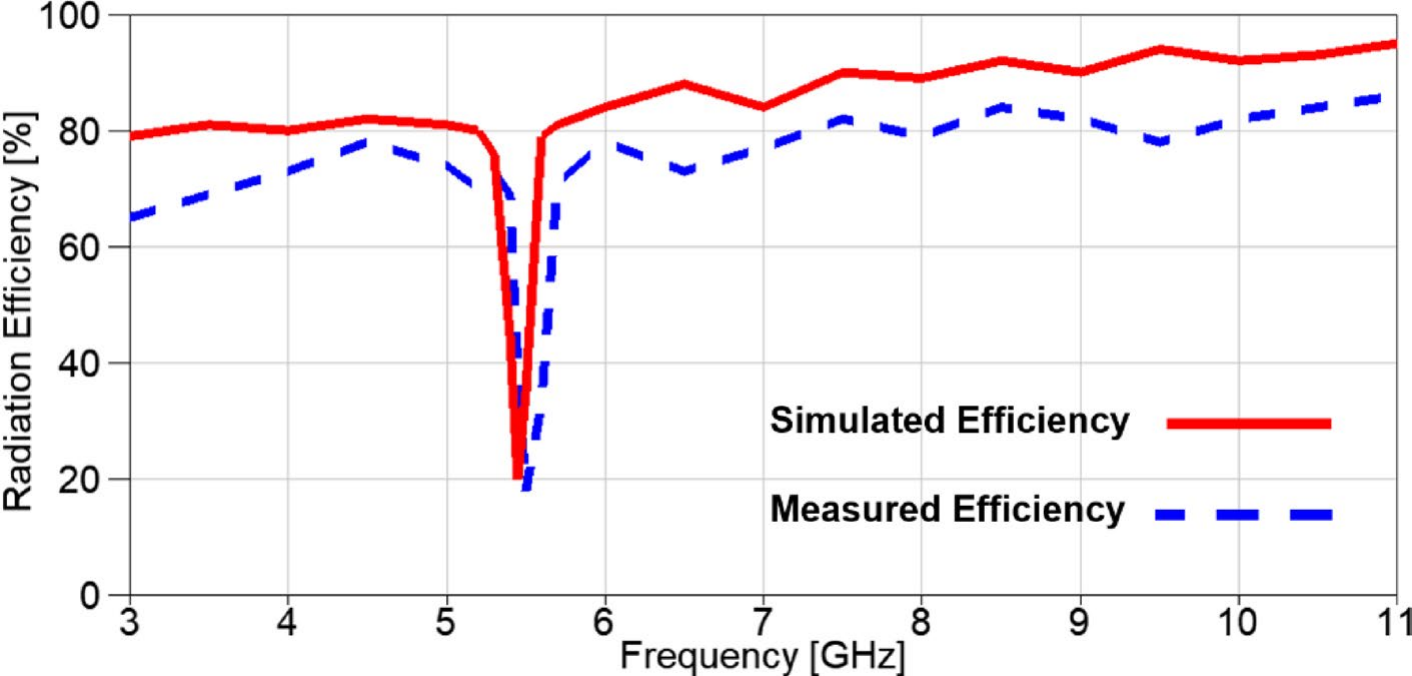
The simulated and measured radiation efficiency of the suggested band-notched MIMO.

The realized gain of the proposed band-notched MIMO antenna is introduced in Figure. 18 and it is clear that there is a negative gain band centered at 5.4 GHz for the simulated gain, while the remaining frequency range is above 0 dB variating from 3 dBi to 6.3 dBi with an average value of 4.1 dBi. On the other hand, the measured realized gain is variating from 1.2 dBi to 4.77 dBi with an average gain value of 2.77 dBi. Furthermore, the radiation efficiency of the proposed MIMO antenna is depicted in Figure. 19 and it can be observed that the simulated efficiency is variating from 79% to 95% with an average value of 85.5%, while the measured efficiency is variating from 65% to 86% with an average value of 76.3%, except the notched bands with lowest values of 20% and 18% for the simulated and measured efficiencies, respectively.

In order to investigate the diversity characteristics of the band-notched MIMO antenna, different parameters such as diversity gain (DG) and envelop correlation coefficient (ECC) need to be evaluated which are mainly based on the isolation between the two ports of the suggested MIMO antenna where ECC is a measure of the correlation between the antenna elements and can be calculated using the following Eqs^33,34^.

3 $$\:ECC={\rho\:}_{e}=\left|{\rho\:}_{12}\right|=\frac{{\left|{S}_{11}^{\mathrm{*}}{S}_{12}+{S}_{21}^{\mathrm{*}}{S}_{22}\right|}^{2}}{\left(1-\left({\left|{S}_{\mathrm{11}}\right|}^{2}+{\left|{S}_{\mathrm{21}}\right|}^{2}\right)\right)\left(1-\left({\left|{S}_{\mathrm{22}}\right|}^{2}+{\left|{S}_{\mathrm{12}}\right|}^{2}\right)\right)}$$

where *ρ*_e_ = *ρ*_12_ are envelop correlation coefficient between the two ports, and *S*_11_, *S*_22_, *S*_12_, *S*_21_ are the scattering parameters of the two ports.

**Fig. 20:**
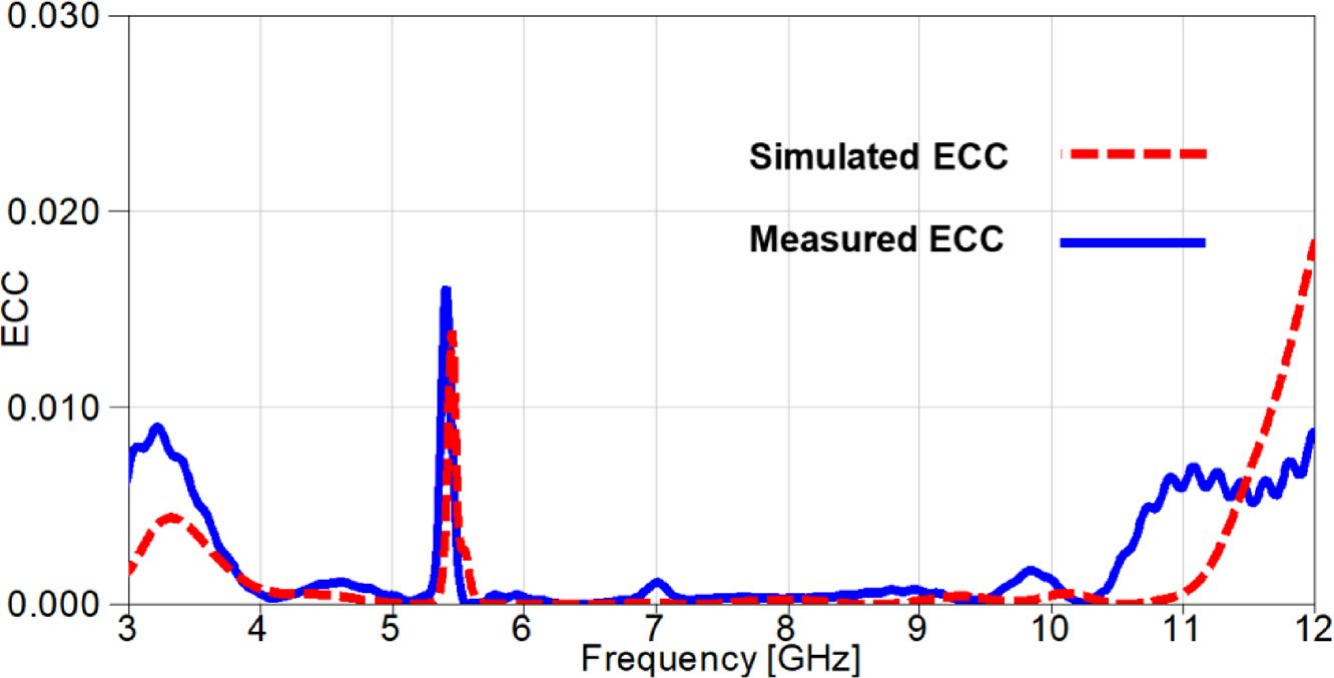
The ECC outcomes of the suggested 2-port MIMO antenna.

The simulated and measured outcomes of the ECC is presented in Figure. 20. It is worth noting that the outcomes are consistent since the higher values of ECC are at the notched-band with a very small deviation. Moreover, all the values are approximately below 0.009, except for the band-notch with a value of 0.016 for the measured case and a value 0.014 for the simulated case. All the previous values of ECC are below the acceptable limit of ECC (≤ 0.5).

DG is another parameter that can be used to investigate the diversity capability of the suggested MIMO antenna and it is directly related to ECC and can be calculated using the following Eqs^33,34^.

4 $$\:DG=10\times\:\sqrt{1-\left|ECC\right|}$$

**Fig. 21:**
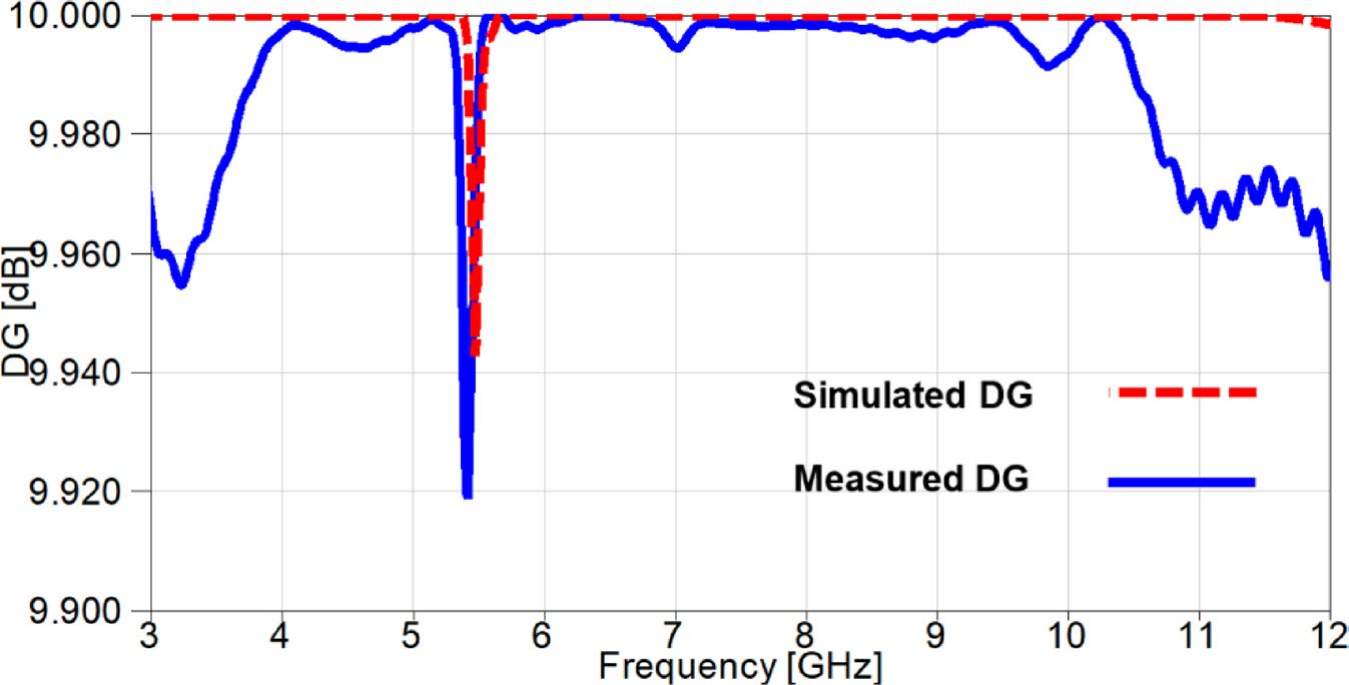
The DG outcomes of the suggested 2-port MIMO antenna.

It can be observed from Figure. 21 that the DG values are above 9.96 dB over the whole achieved band, except for the notched band with a value of 9.945 dB for the simulated case and a value of 9.92 for the measured case.

## Reconfigurable 2-port MIMO antenna layout

**Fig. 22:**
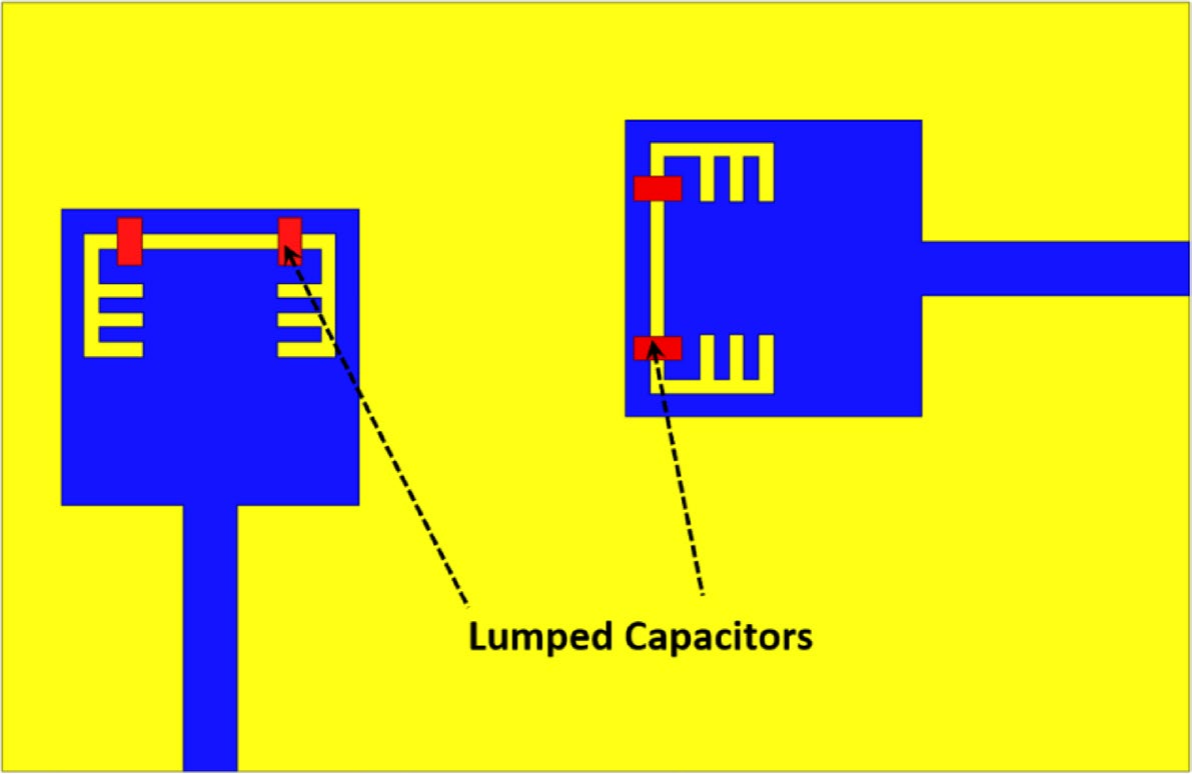
Reconfigurable Band-notched 2-port MIMO UWB antenna.

In the previous section, the 2-port MIMO antenna succeeded to provide an UWB behavior from 3 to 10.6 GHz, except at 5.4 GHz due to the etched band-rejection structures from the two radiators, which is basically used to mitigate interference with WLAN applications. In this section, a frequency reconfiguration of the band-notched behavior of the proposed MIMO antenna will be presented and this can be accomplished by embedding two pairs of lumped capacitors on the two band-rejection structures as depicted in Figure. 22. The main target is to provide a controllability on the behavior of the band-notched structure by electronically tune the notched frequency from one application to another for interference mitigation purpose, and in our case, we can specifically switch between WLAN centered at 5.4 GHz to WiMAX centered at 3.5 GHz by using two pairs of lumped capacitors.

**Fig. 23:**
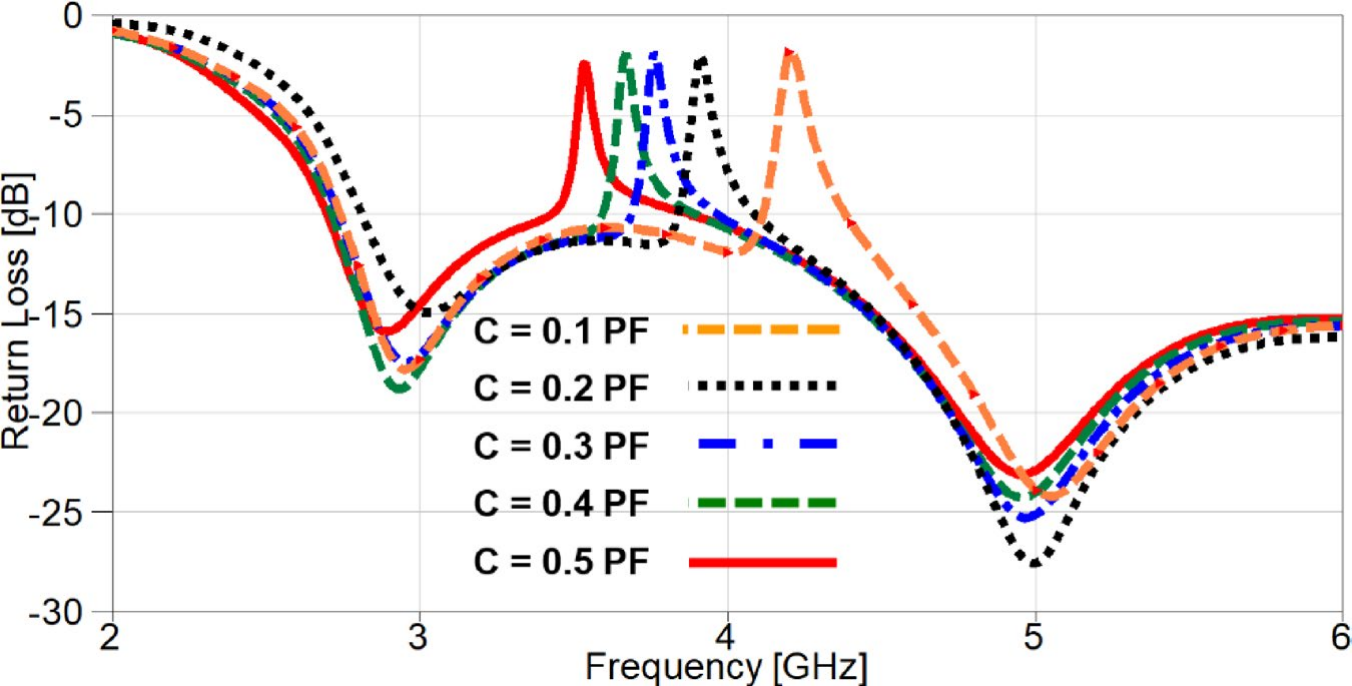
The effect of changing capacitor values on S_11_.

**Fig. 24:**
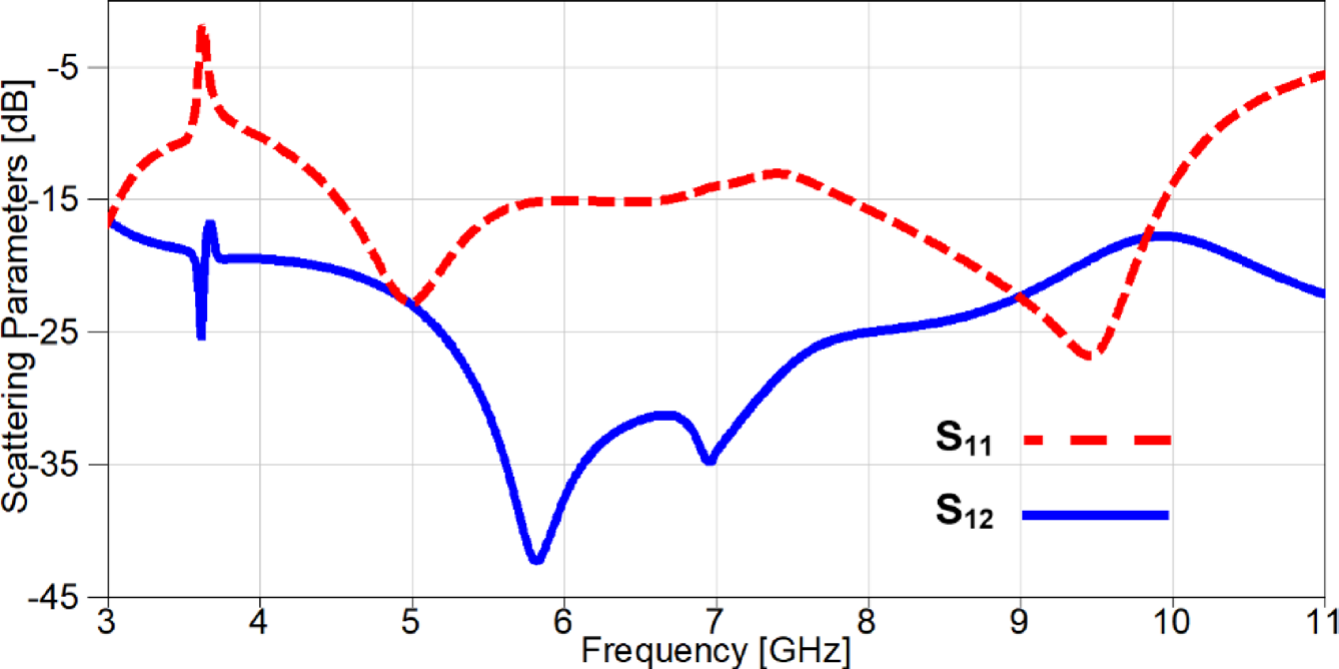
The scattering parameters of the reconfigurable 2-port MIMO antenna.

A parametric study on the values of lumped capacitors is carried out in order to investigate the effect of changing capacitance on the resonance frequency of the notched-band. It can be noticed from Figure. 23 that the values are selected from 0.1 PF to 0.5 PF and the resonance is moving from 4.2 GHz to 3.53 GHz, respectively. The simulated S_11_ and S_21_ of the reconfigurable MIMO antenna after carrying out the parametric study to select the appropriate capacitor values for the desired performance, is presented in Figure. 24. It is clear the band-notch is resonating at 3.53 GHz for interference mitigation with WiMAX applications, also the isolation between the two ports is more than 17 dB over the achieved frequency band reaching up to 47 dB at 5.85 GHz.

**Fig. 25:**
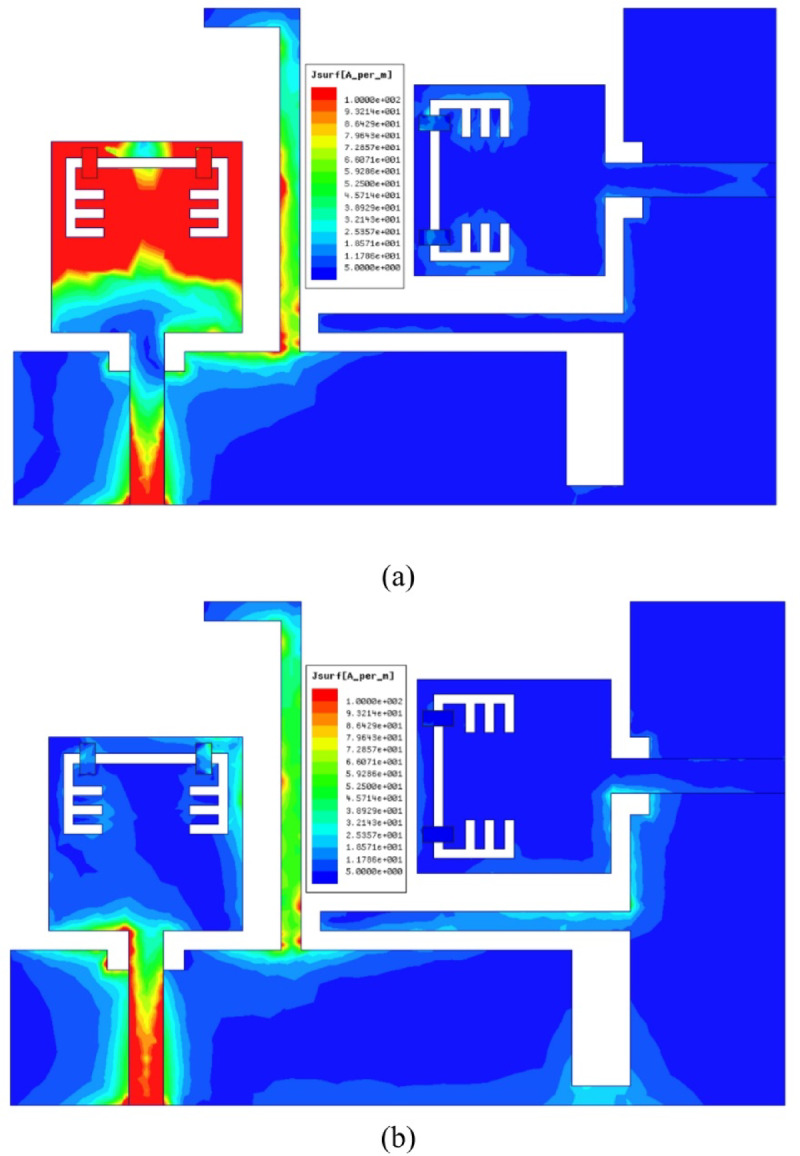
The current distribution of the reconfigurable band-notched MIMO at two different frequencies (**a**) at 3.53 GHz (**b**) at 4 GHz.

The simulation results of the current distribution over the reconfigurable MIMO antenna in order to validate the desired behaviour and it can be noticed form Figure. 25 (a) that the current is collected around the band-notched structure and saturated over the lumped capacitors when the antenna is simulated at 3.53 GHz confirming the antenna can’t radiate when excited with 3.53 GHz RF signal, also the neighbor element didn’t receive any current due to the isolation stubs in the ground plane. On the contrary, the antenna can radiate efficiently when excited with any other frequency inside the achieved band, except 3.53 GHz. In Figure. 25 (b), the antenna is excited with 4 GHz RF signal (0.7 GHz difference to confirm the selective property of the suggested antenna) and it is clear that the current is uniformly distributed, and very small amount of current can be transferred to the neighbor element due to the distinct isolation between ports.

**Fig. 26:**
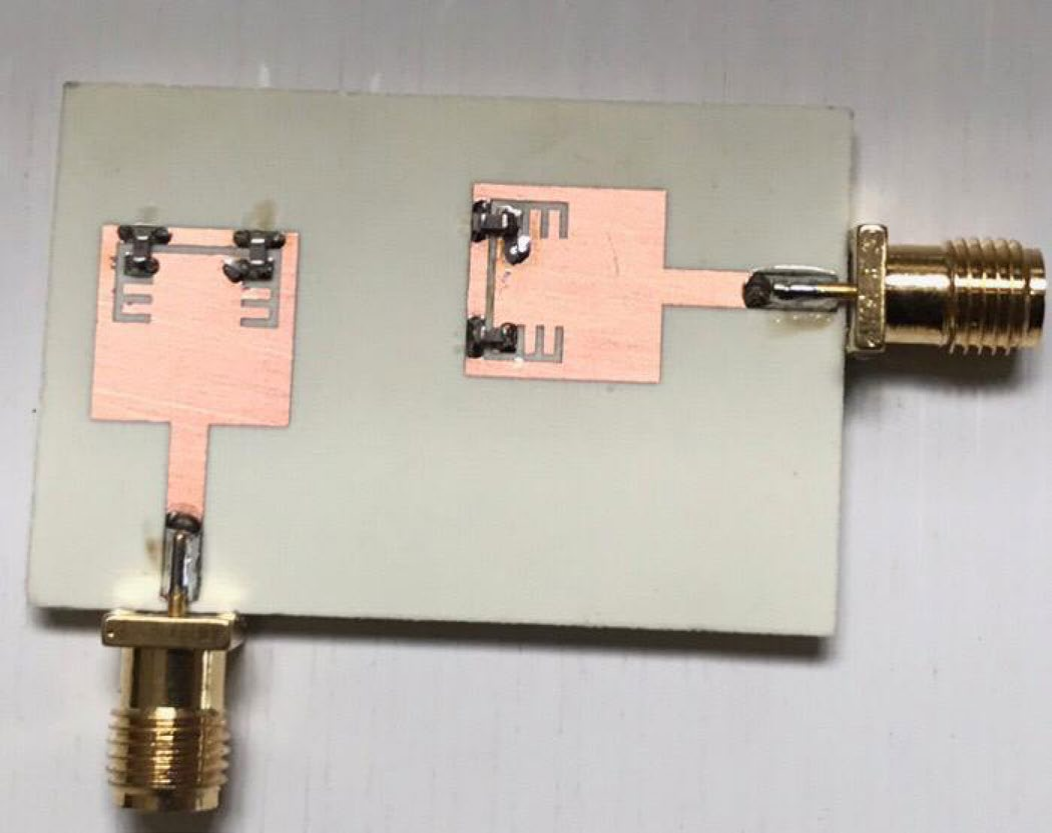
The fabricated photograph of the reconfigurable 2-port MIMO antenna.

In order to achieve the reconfigurability for the suggested 2-port MIMO antenna, two pairs of lumped capacitors of value 0.5 PF are embedded on the previously fabricated antenna shown in Figure.15 to reject the WiMAX band. Figure. 26 depicts the fabricated antenna after welding the 4 lumped capacitors and then I will be tested to validate the simulation results.

**Fig. 27:**
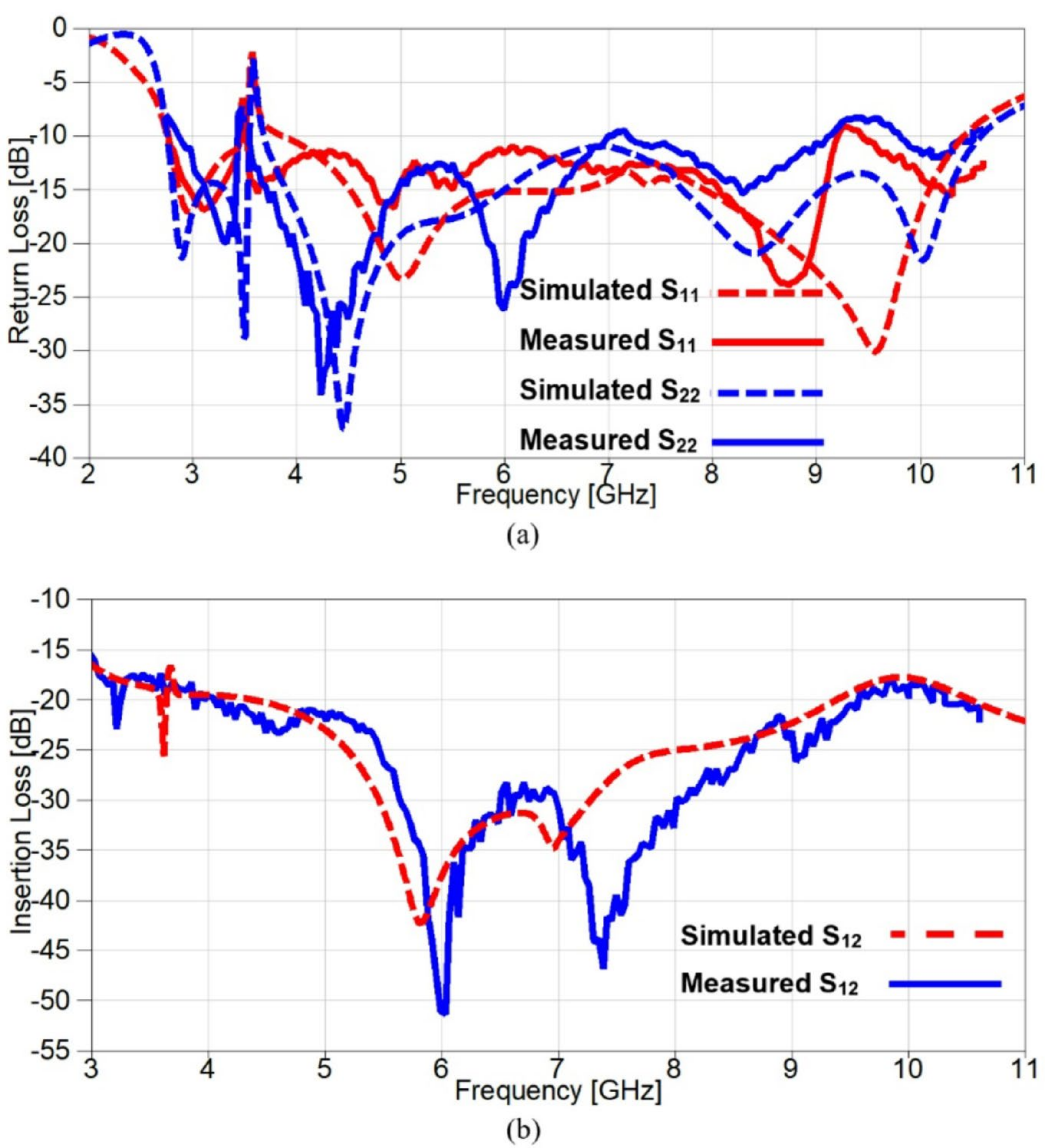
The scattering parameters of the reconfigurable band-notched 2-port MIMO antenna (**a**) Return loss (**b**) Insertion loss.

Figure. 27 introduces a comparison between simulation and experimental outcomes, it is evident from the obtained results in Figure. 27 (a) that the measured outputs mimic the simulated ones since both results give a notch above − 10 dB at 3.5 GHz with a small deviation between the two notches at WiMAX frequency band. Furthermore, the return loss results are below − 10 dB from 2.6 GHz to 10.5 GHz, but a small part of measured S_22_ is above − 10 dB from 9.15 to 9.8 GHz and all these small discrepancies are due to the fabrication tolerance in addition to the embedded capacitors. The isolation between elements is investigated in Figure. 27 (b) and a good consistency between both results is illustrated clearly since the mutual coupling is below − 17 dB over the achieved frequency range, also the insertion losses reach up to −42 dB and − 51.5 dB for simulated and measured outcomes, respectively.

**Fig. 28:**
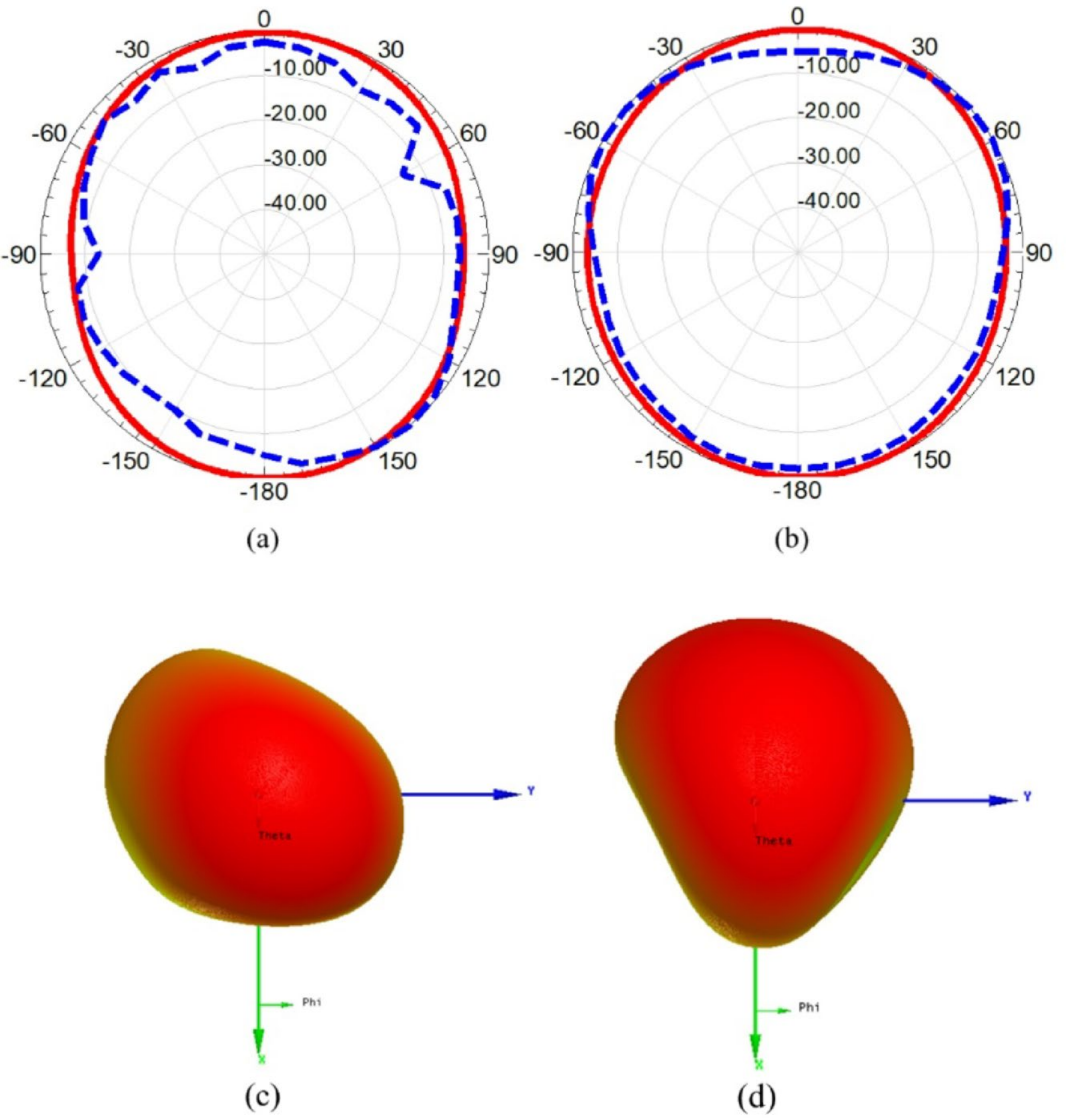
The 2-D simulated (solid) and measured (dashed) radiation patterns, and 3-D gain pattern of the reconfigurable MIMO antenna at 4 GHz (**a**) x-z plane (**b**) at y-z plane (**c**) 3-D pattern for port 1 at x-y plane (d) 3-D pattern for port 2 at x-y plane.

The upcoming part of the paper will introduce the radiation characteristics of the suggested reconfigurable 2-port MIMO antenna. Figure. 28 (a) and (b) shows the 2-D radiation pattern in E and H planes. It can be noticed that the pattern is nearly omnidirectional in both planes that cope the needs of modern wireless devices with good agreement between simulation and experimental outcomes. Moreover, the 3-D radiation patterns in x-y plane for each excited port are shown in the Figure. 28 (c) and (d) and it is evident that the suggested MIMO antenna provides a pattern diversity with an approximate orthogonal beam directions due to the orthogonal arrangement of the two elements confirming the low-level of cross polarization as well as the independency of each element for radiating/receiving EM waves.

**Fig. 29:**
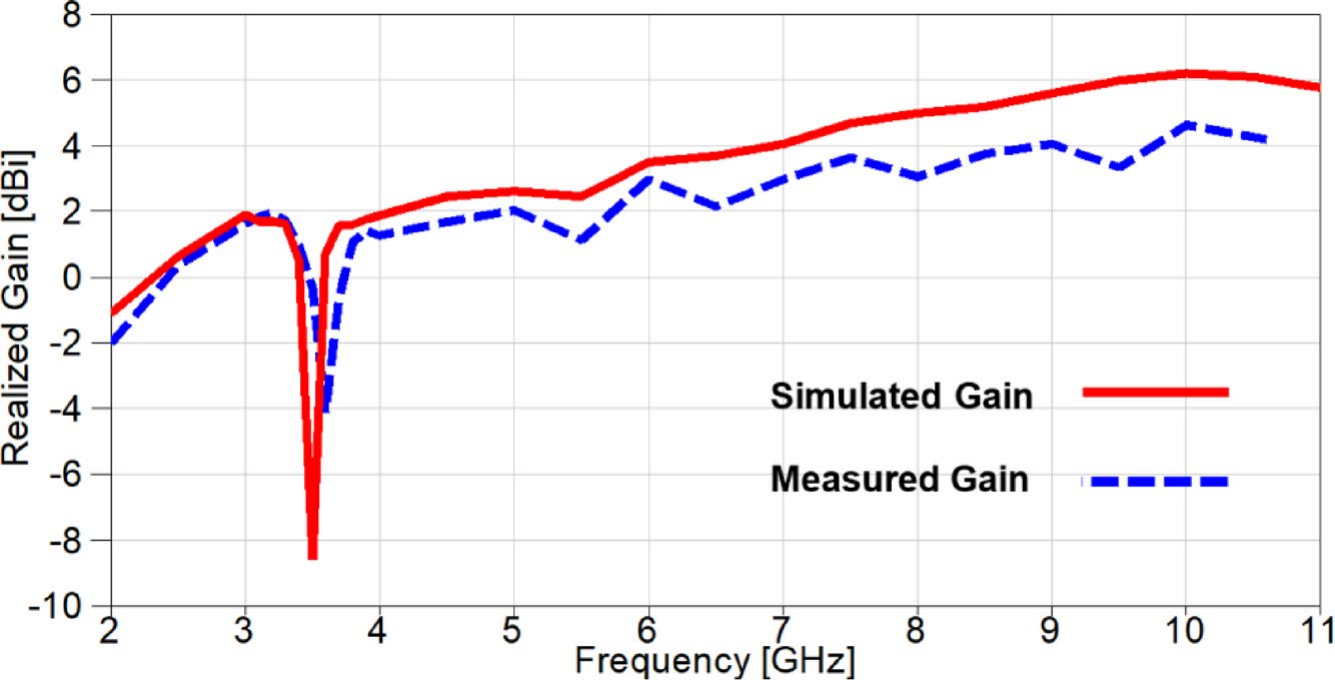
The simulated and measured realized gain of the reconfigurable band-notched MIMO.

The realized gain of the reconfigurable band-notched 2-port MIMO antenna is demonstrated in Figure. 29 and it can be noticed that the gain increases dramatically over the achieved band, except a band-stop band at the WiMAX frequency band centered at 3.5 GHz. The measured outcome agreed with the simulated one, but a small discrepancy between results because of fabrication tolerance and measurement setup inside the anechoic chamber. The simulated gain is varied from 0.6 dBi to 6.1 dBi with an average value of 3.1 dBi. On the contrary, the measured realized gain is varied from 0.35 dBi to 4.62 dBi with an average gain value of 2.15 dBi.

**Fig. 30:**
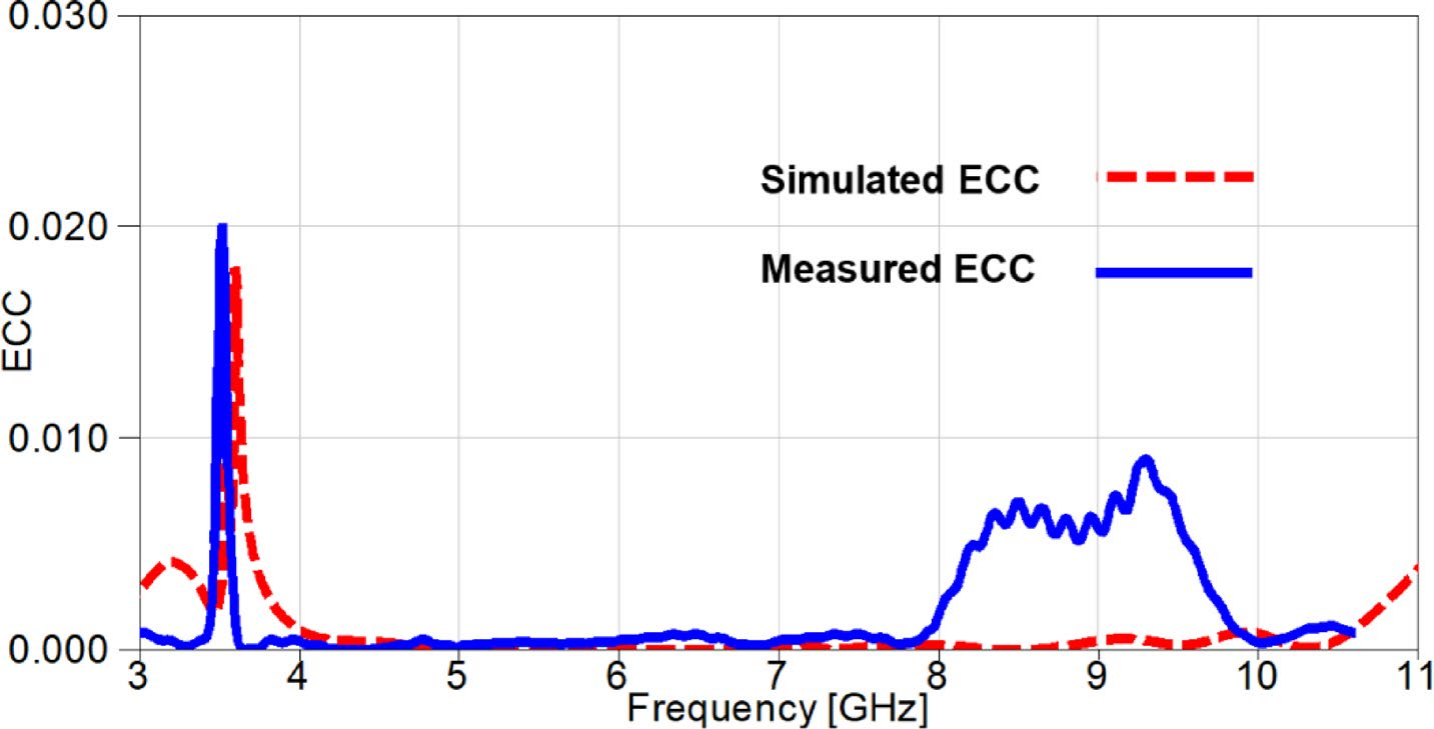
The ECC outcomes of the reconfigurable MIMO antenna.

**Fig. 31:**
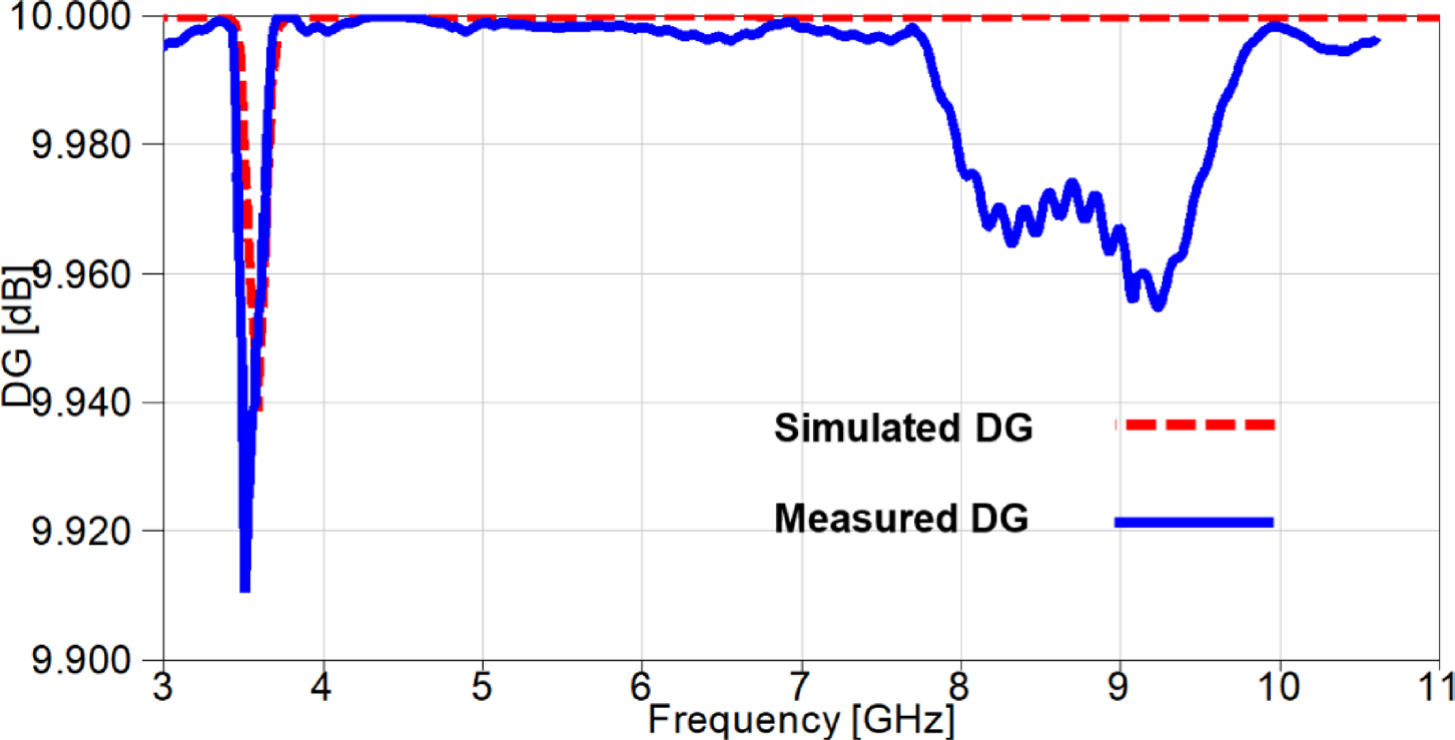
The DG outcomes of the reconfigurable MIMO antenna.

The outcomes of diversity parameters are presented in Figs. 30 and 31 to investigate the MIMO performance of the reconfigurable UWB antenna. The ECC values in Figure. 30 demonstrate the good diversity of the suggested antenna since the values are below 0.02 at the notched-band, whereas the remaining band results are below 0.009. Additionally, The DG is directly related to ECC through Eq. (4) and its values are approximately underneath 10 dB with a value of 9.91 dB at 3.5 GHz and values greater than 9.955 dB for the remaining achieved frequency range as depicted in Figure. 31.

**Fig. 32:**
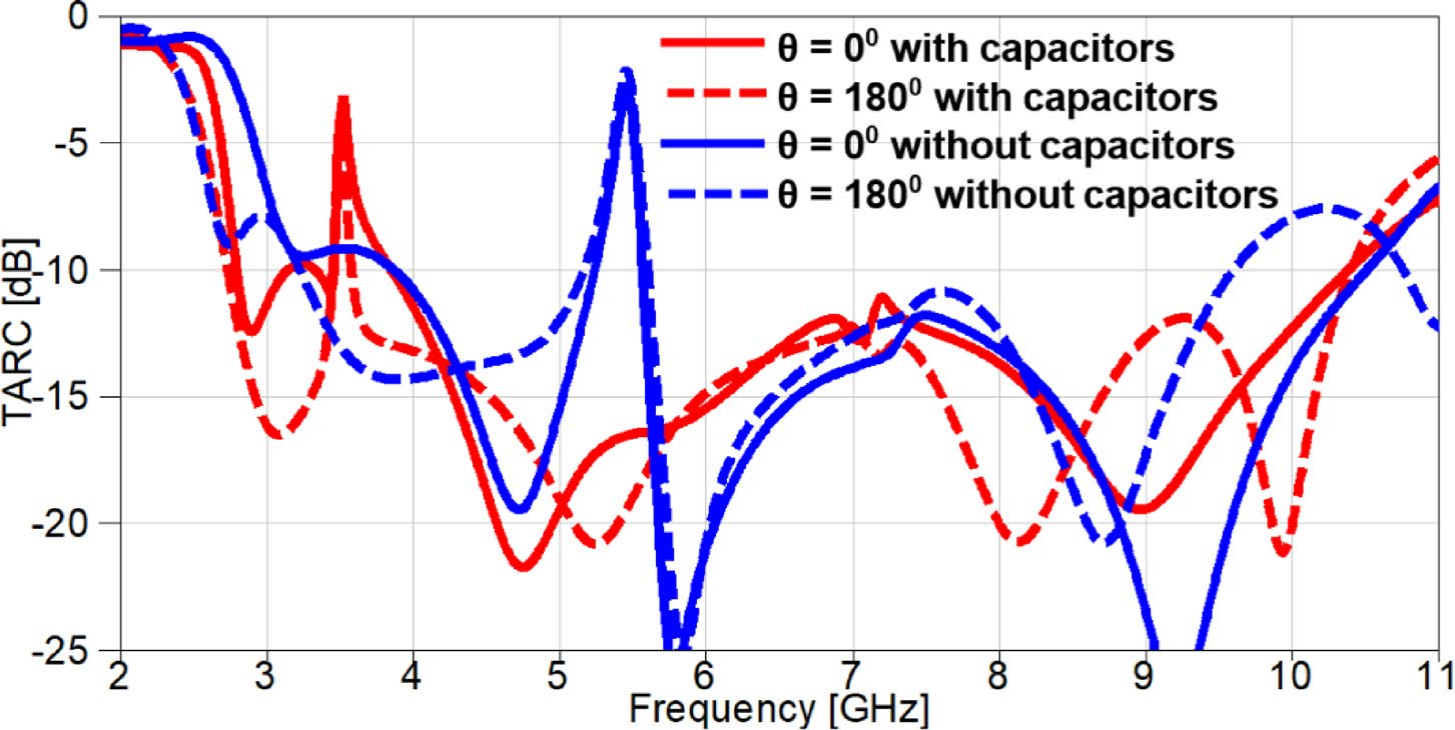
The TARC outcomes of the suggested MIMO antenna.

The MIMO performance can be evaluated using total active reflection coefficient (TARC) and can be calculated using S-parameters. TARC can be calculated and extracted using Eq. (5)^35^.

5 $$\:TARC=\sqrt{\frac{{\left|{S}_{11}+{S}_{12}{e}^{j\theta\:}\right|}^{2}+{\left|{S}_{21}+{S}_{22}{e}^{j\theta\:}\right|}^{2}}{2}}$$

where *S*_11_, *S*_22_, *S*_12_, *S*_21_ are the scattering parameters of the two ports, and θ is the phase angle. The TARC results of the MIMO antenna at 0^o^ and 180^o^ phases for both cases (with/without lumped capacitors) are shown in Fig. 32. The TARC values ​​are less than − 10 dB in the desired frequency band at both phases except the notched bands (5.4 GHz without capacitors and 3.53 GHz with capacitors), which is very consistent with the MIMO performance requirements. A small discrepancy can be observed for the case of MIMO antenna without using lumped capacitors at 180^0^ where the TARC values are above − 10 dB from 9.5 GHz till the end of desired frequency band.

In order to assure the competitive advantages of the suggested reconfigurable band-notched MIMO with the state-of-the-art, a comparison with recently published papers is tabulated in Table 1. The comparison in Table 1 is introduced in terms of number of elements, size, frequency band, realized gain, isolation and ECC, and it can be demonstrated that the suggested work has significant advantages compared to existing literature across key performance metrics. A standout feature is the exceptionally low ECC (less than 0.009) which significantly outperforms most of the references, except^28–31^, establishing a superior benchmark for isolation and diversity performance in MIMO design, in addition to the acceptable realized gain performance reaching up to 4.7 dBi experimentally. Architecturally, our model achieves a remarkably compact size of 40 × 26 mm^2^ compared to most of MIMO models in the table, while utilizing a significantly thinner thickness of 0.8 mm, surpassing the profile constraints of all referenced models, except^21^. Furthermore, the choice of 4-lumped capacitors as actuators offers a distinct approach to reconfigure the desired frequency, diverging from the prevalent use of PIN diodes and varactor diodes in^21–31^, potentially leading to simplified biasing and integration. It can be observed that the suggested reconfigurable MIMO antenna outperforms the listed antennas in various parameters which confirms the eligibility of the reconfigurable MIMO antenna for wide range of wireless communication applications.

**Table 1:** The suggested reconfigurable antenna vs. recently published designs.

Ref.	No of elements	ε_*r*_/thickness (mm)	Recon. Freq. [GHz]	Realized Gain (dBi)	Isolation (dB)	ECC	Actuators	Size (mm^2^)
^16^	1	4.4/1.6	5.6/3.5	0.3–4.3	–	–	2 Lumped capacitors	32 × 32
^21^	1	11.9/0.4	35	5	–	–	2 MEMS	3.7 × 5.2
^22^	1	3.38/0.813	1.75/2.16/2.8/3	4.32/4.65/4.54/5.05	–	–	4 Varactor diodes	80 × 80
^23^	2	3.55/1.52	0.8–0.98/1.65–2.2	3.44	≥ 20	≤ 0.054/0.044	4 Varactor diodes	100 × 63
^24^	1	3.38/0.813	1.5–3.5/1.6/2/2.5/3	2	–	–	4 PIN diodes	60 × 60
^25^	1	4.4/1.6	2.5–4.2/6–7.2	4.01/4.60	–	–	3 PIN diodes	23 × 31
^26^	2	4.4/1.6	4.61–5.53/1.67–1.9	6.63/4.41	≥ 42.6/26.5	≤ 0.031/0.253	1 PIN diode	48 × 24
^27^	2	4.4/1.6	4.9–6.3	2–5.5	≥ 17	≤ 0.15	4 PIN diodes	50 × 25
^28^	2	3.38/1.52	2.2–2.7/3.3–4.02	3.7/4.2	≥ 12	≤ 0.0056/0.0009	4 PIN diodes	120 × 60
^29^	2	4.4/1.6	5.15–5.82	1–2.5	≥ 20	≤ 0.001	2 PIN diodes	40 × 23
^30^	4	4.4/1.6	3.1–12.1/8.3–11.6/2.9–6.1,8.4–10.5	5.7/5.5/5	≥ 18.6	≤ 0.006	8 PIN diodes	50 × 50
^31^	2	4.4/1.6	3.5/4.5/7.5	1.15 − 5.23	≥ 32	≤ 0.001	6 PIN diodes	60 × 50
This work	2	3.66/0.8	5.4/3.5	1.2–4.7	≥ 17	≤ 0.009	4 Lumped capacitors	40 × 26

## Conclusion

A 2-port reconfigurable MIMO antenna has been introduced in this paper to switch between two notched-bands for interference mitigation with WiMAX/WLAN at 3.5/5.4 GHz. The antenna has been designed on RO4350 substrate with dielectric constant of 3.66 and loss tangent of 0.004 with an area of 40 × 26 mm^2^. The suggested MIMO antenna has been fabricated and tested to investigate its impedance and radiation characteristics. Thereafter, two pairs of lumped capacitors have been embedded on the resonators to achieve the band-notched reconfigurability. The reconfigurable antenna has been tested and a good consistency between simulated and measured impedance matching has been obtained. The isolation between elements is more than 17 dB which in turn resulted in a distinct diversity performance in terms of ECC less than 0.009 and DG greater than 9.955 dB. the suggested antenna can be considered an outstanding candidate for various wireless applications due to its competitive advantages with modern antennas.

## Data Availability

All data generated or analyzed during this study are included in this article.

## References

[1] Nagy, L. Microstrip antenna development for radar sensor. Sensors 23, 2 : 909. (2023). 10.3390/s23020909 10.3390/s23020909 PMC9866705 36679704

[2] Kadam, P. A., Amit, A. & Deshmukh Designs of regular shape microstrip antennas backed by bow-tie shape ground plane for enhanced antenna characteristics. *AEU-International J. Electron. Commun.* **137**, 153823. 10.1016/j.aeue.2021.153823 (2021).

[3] Zhao, Y., Li, Y. & Shi, W. and Wenhua Yu. Mutual coupling reduction between patch antenna and microstrip transmission line by using defected isolation wall. *Appl. Comput. Electromagnet. Soc. J. (ACES). ***34** (1), 100–106. (2019).

[4] Gaya, S., Sokunbi, O., Hamza, A., Sharif, I. M. & Sheikh and Hussein Attia. Multiple-input‐multiple‐output antenna with pattern reconfiguration and correlation reduction for WLAN applications. Engineering reports. **2 **(12), e12272. (2020). 10.1002/eng2.12272

[5] Shirpay, A., Rohaninezhad, M. R., Tavakoli, M. & Zarezadeh, E. Reduction of mutual coupling in a microstrip array antenna with circular polarization in the C-frequency band using a combination of DGS and EBG methods. *Eng. Res. Express*. **5** (1), 015033. 10.1088/2631-8695/aca6c1 (2023).

[6] Jemaludin, N. H. et al. Imran Mohd Ibrahim, and Zahriladha Zakaria. A comprehensive review on MIMO antennas for 5G smartphones: mutual coupling techniques, comparative studies, SAR analysis, and future directions. *Results Eng.* 102712. 10.1016/j.rineng.2024.102712 (2024).

[7] Elabd, R. H. & Al-Gburi. Super-compact 28/38 ghz 4-port MIMO antenna using metamaterial-inspired EBG structure with SAR analysis for 5G cellular devices. *J. Infrared Millim. Terahertz Waves*. **45** (1), 35–65. 10.1007/s10762-023-00959-6 (2024).

[8] Zhu, L. & Liu, N. Multimode resonator technique in antennas: a review. *Electromagn. Sci.* **1** (1), 1–17. 10.23919/emsci.2022.0004 (2023).

[9] Esmail, B. A. & Slawomir Koziel. High isolation metamaterial-based dual-band MIMO antenna for 5G millimeter-wave applications. *AEU-International J. Electron. Commun.* **158**, 154470. 10.1016/j.aeue.2022.154470 (2023).

[10] Ali, W. A. E., Mohamed, I., Ashraf & Mohammad, A. Salamin. A dual-mode double-sided 4× 4 MIMO slot antenna with distinct isolation for WLAN/WiMAX applications. *Microsyst. Technol.* **27**, 967–983. 10.1007/s00542-020-04984-6 (2021).

[11] Elabd, R. H. Al-Gburi. SAR assessment of miniaturized wideband MIMO antenna structure for millimeter wave 5G smartphones. *Microelectron. Eng.* **282**, 112098. 10.1016/j.mee.2023.112098 (2023).

[12] Thakur, E., Gupta, A., Abdulhameed, M. K., Khaleel, A. D. & Ahmed Jamal Abdullah Al-Gburi. Microstrip antenna with two elements and defected ground structure for 5G mobile applications at 28/38 ghz. *Progress Electromagnet. Res. C*. **146**10.2528/PIERC24062403 (2024).

[13] Hussain, M. Single iterated fractal inspired UWB antenna with reconfigurable Notch bands for compact electronics. *Heliyon.* **9** (11), e21419.10.1016/j.heliyon.2023.e21419 (2023). 37954332 10.1016/j.heliyon.2023.e21419 PMC10637985

[14] Mukherjee, S. et al. Notch band characteristics improvement of a printed ultra wideband antenna by embedding frequency selective surface. *AEU-International J. Electron. Commun.* **178**, 155276. 10.1016/j.aeue.2024.155276 (2024).

[15] Abbas, A. et al. Highly selective multiple-notched UWB-MIMO antenna with low correlation using an innovative parasitic decoupling structure. *Eng. Sci. Technol. Int. J.* **43**, 101440. 10.1016/j.jestch.2023.101440 (2023).

[16] Ali, W. A. E., Ahmed, A. & Ibrahim Tunable band-notched UWB antenna from WLAN to wimax with open loop resonators using lumped capacitors. *Appl. Compu Electro Soc. J. (ACES)*. 603–609. 10.47037/2018.ACES.J.330618 (2018).

[17] Salamin, M., Ahmad, W. A. & Zugari, A. Design and analysis of a miniaturized band-notched planar antenna incorporating a joint DMS and DGS band-rejection technique for UWB applications. *Microsyst. Technol.* **25** : 3375–3385. 10.1007/s00542-018-4183-9 (2019).

[18] Ali, W. A., Ellatif, Rana, M. A. & Moniem Frequency reconfigurable triple band-notched ultra-wideband antenna with compact size. Progress. *Electromagnet. Res. C*. **73**, 37–46. 10.2528/PIERC17021001 (2017).

[19] Ibrahim, A., Ali, W. & Machac, J. UWB monopole antenna with band notched characteristics mitigating interference with WiMAX. Radioengineering 26, no. 2 : 438–443. (2017). 10.13164/re.2017.0438

[20] Hussain, M., Ali, E. M., Awan, W. A., Hussain, N. & Alibakhshikenari, M. Virdee, and Francisco Falcone. Electronically reconfigurable and conformal triband antenna for wireless communications systems and portable devices. *Plos One*. **17** (12), e0276922 (2022). 36454808 10.1371/journal.pone.0276922 PMC9714694

[21] Deng, Z. & Wang, Y. and Chengqi Lai. Design and analysis of pattern reconfigurable antenna based on RF MEMS Switches. Electronics 12, no. 14 : 3109. (2023). 10.3390/electronics12143109

[22] Abdelghany, M. A., Wael, A. E., Ali, H. A., Mohamed & Ahmed, A. Ibrahim. Filtenna with Frequency Reconfigurable Operation for Cognitive Radio and Wireless Applications. Micromachines 14, no. 1 : 160. (2023). 10.3390/mi14010160 10.3390/mi14010160 PMC9862267 36677220

[23] Shruthi, G. & Microwaves Choukiker Yogesh Kumar. Dual-band frequency‐reconfigurable MIMO PIFA for LTE applications in mo-bile hand‐held devices. IET *Antennas Propag.* **14**, 5 : 419–427. 10.1049/iet-map.2019.0878 (2020).

[24] Ibrahim, A. A., Wael, A. E., Ali, M., Alathbah & Mohamed, H. A. A frequency reconfigurable folded antenna for cognitive radio communication micromachines 14, 3: 527. (2023). 10.3390/mi14030527. https://doi.org/10.3390/mi14030527. 10.3390/mi14030527 PMC10056032 36984934

[25] Iqbal, A. et al. Frequency and pattern reconfigurable antenna for emerging wireless communication systems electronics 8, 4: 407. (2019). 10.3390/electronics8040407

[26] Islam, H. et al. A frequency reconfigurable MIMO antenna with Bandstop filter decoupling network for cognitive communication sensors 22, no. **18**: 6937. (2022). 10.3390/s22186937 10.3390/s22186937 PMC9501463 36146286

[27] Khan, M. et al. Ultra-Compact reconfigurable band reject UWB MIMO antenna with four radiators electronics 9, no. 4: 584. (2020). 10.3390/electronics9040584

[28] Pant, A., Singh, M. & Manoj Singh Parihar A frequency reconfigurable/switchable MIMO antenna for LTE and early 5G applications. *AEU-international J. Electron. Commun.* **131**, 153638. 10.1016/j.aeue.2021.153638 (2021).

[29] Quddus, A., Saleem, R., Arain, S., Hassan, S. R. & Farhan Shafique, M. Electronically reconfigurable WLAN band-notched MIMO antenna for ultra-wideband applications. *Appl. Comput. Electromagnet. Soc. J. (ACES)*. 1108–1111. 10.47037/2021.ACES.J.360821 (2021).

[30] Durukan, T. & Altuncu, Y. A compact 4× 4 reconfigurable MIMO antenna design with adjustable suppression of certain frequency bands within the UWB frequency range. *AEU-International J. Electron. Commun.* **170**, 154848. 10.1016/j.aeue.2023.154848 (2023).

[31] Nej, S., Ghosh, A., Kumar, J. & Das, S. Ultra-wideband MIMO antenna with reconfigurable band Notch characteristics and improved isolation. *AEU-International J. Electron. Commun.* **170**, 154849. 10.1016/j.aeue.2023.154849 (2023).

[32] Awan, W., Abbas, A., Zaidi, M., Hussain, N. & Hussain and Ikram Syed. The design of a wideband antenna with Notching characteristics for small devices using a genetic algorithm mathematics 9, no. **17**: 2113. (2021). 10.3390/math9172113

[33] Ali, W. A. E., Ahmed, A., Ibrahim & Ashraf, E. Ahmed. Dual-Band millimeter wave 2× 2 MIMO slot antenna with low mutual coupling for 5G Networks. Wireless personal communications 129, 4 : 2959–2976. (2023). 10.1007/s11277-023-10267-w

[34] Ibrahim, A. A., Wael, A. E., Ali, M., Alathbah & Sabek, A. R. Four-Port 38 GHz MIMO antenna with high gain and isolation for 5G wireless networks sensors 23, no.**7** : 3557. 10.3390/s23073557(2023). 10.3390/s23073557 PMC10099217 37050618

[35] Urimubenshi, F., Dominic, B. O. & Konditi Jean de dieu Iyakaremye, Pierre Moukala Mpele, and Augustin Munyaneza. A novel approach for low mutual coupling and ultra-compact two Port MIMO antenna development for UWB wireless application. *Heliyon.* **8** (3), e09057. 10.1016/j.heliyon.2022.e09057(2022). 10.1016/j.heliyon.2022.e09057 PMC8914126 35284684

